# CK2 is a key regulator of SLC4A2-mediated Cl^−^/HCO_3_^−^ exchange in human airway epithelia

**DOI:** 10.1007/s00424-017-1981-3

**Published:** 2017-04-28

**Authors:** Salam H. Ibrahim, Mark J. Turner, Vinciane Saint-Criq, James Garnett, Iram J. Haq, Malcolm Brodlie, Chris Ward, Christian Borgo, Mauro Salvi, Andrea Venerando, Michael A. Gray

**Affiliations:** 1 Institute for Cell & Molecular Biosciences, The Medical School, Newcastle University, Framlington Place, Newcastle upon Tyne, NE2 4HH UK; 2 Department of Basic Science, College of Veterinary Medicine, University of Sulaimani, Sulaimani City, Kurdistan Region Iraq; 3 Department of Physiology, McGill University, Montreal, Canada; 4 Institute of Cellular Medicine, Newcastle University, Newcastle upon Tyne, UK; 5 Immunology & Respiratory Diseases Research, Boehringer Ingelheim Pharma GmbH & Co. KG, Biberach an der riss, Germany; 6 Department of Biomedical Sciences, CNR Institute of Neuroscience, University of Padova, Padova, Italy

**Keywords:** CK2, SLC4A2, Anion transport, HCO_3_^−^, Airway epithelia

## Abstract

Transepithelial bicarbonate secretion by human airway submucosal glands and surface epithelial cells is crucial to maintain the pH-sensitive innate defence mechanisms of the lung. cAMP agonists stimulate HCO_3_^−^ secretion via coordinated increases in basolateral HCO_3_^−^ influx and accumulation, as well as CFTR-dependent HCO_3_^−^ efflux at the luminal membrane of airway epithelial cells. Here, we investigated the regulation of a basolateral located, DIDS-sensitive, Cl^−^/HCO_3_^−^ exchanger, anion exchanger 2 (AE2; SLC4A2) which is postulated to act as an acid loader, and therefore potential regulator of HCO_3_^−^ secretion, in human airway epithelial cells. Using intracellular pH measurements performed on Calu-3 cells, we demonstrate that the activity of the basolateral Cl^−^/HCO_3_^−^ exchanger was significantly downregulated by cAMP agonists, via a PKA-independent mechanism and also required Ca^2+^ and calmodulin under resting conditions. AE2 contains potential phosphorylation sites by a calmodulin substrate, protein kinase CK2, and we demonstrated that AE2 activity was reduced in the presence of CK2 inhibition. Moreover, CK2 inhibition abolished the activity of AE2 in primary human nasal epithelia. Studies performed on mouse AE2 transfected into HEK-293T cells confirmed almost identical Ca^2+^/calmodulin and CK2 regulation to that observed in Calu-3 and primary human nasal cells. Furthermore, mouse AE2 activity was reduced by genetic knockout of CK2, an effect which was rescued by exogenous CK2 expression. Together, these findings are the first to demonstrate that CK2 is a key regulator of Cl^−^-dependent HCO_3_^−^ export at the serosal membrane of human airway epithelial cells.

**Electronic supplementary material:**

The online version of this article (doi:10.1007/s00424-017-1981-3) contains supplementary material, which is available to authorized users.

## Introduction

The secretion of bicarbonate (HCO_3_^−^) by epithelial cells is essential for maintaining the normal function of many epithelial tissues primarily due to its ability to act as a biological buffer and therefore a key regulator of extracellular pH [22]. In the human airways, HCO_3_^−^ plays a major role in the innate defence of the lungs to inhaled pathogens. Transepithelial secretion of HCO_3_^−^ and Cl^−^ from serous cells of the submucosal glands, as well as surface epithelial cells, drives isosmotic water secretion, and this HCO_3_^−^-rich fluid is an important component of the perciliary layer (PCL) of the airway surface liquid (ASL) which lines the conducting airways [4]. The depth of the PCL is important for efficient mucociliary clearance [5, 62] while maintaining the correct ASL pH is vital for efficient bacterial killing by pH-dependent antimicrobials as well as reducing mucus viscosity [17, 44, 53]. In addition, HCO_3_^−^ allows for efficient solubilization and transportation of airway mucus [47]. Therefore, HCO_3_^−^ secretion enables pH-sensitive components of the innate defence mechanisms of the lung to function efficiently.

Transepithelial HCO_3_^−^ secretion is a two-stage process that involves (i) import across the basolateral membrane and accumulation above electrochemical equilibrium and (ii) exit across the apical (serosal) membrane down its electrochemical gradient. It is well established that cAMP-dependent stimulation of the cystic fibrosis transmembrane conductance regulator (CFTR) is crucial for HCO_3_^−^ exit across the luminal membrane in human airway epithelia [11, 14, 18, 29, 54, 56]. Loss of functional CFTR causes cystic fibrosis (CF), an autosomal recessive disorder characterized by the accumulation of thick, sticky mucus, bacterial colonization and chronic inflammation of the airways [34]. Impaired cAMP-stimulated HCO_3_^−^ secretion has been reported in a wide range of CF tissues [6], and the ASL of CF patients is more acidic than that of healthy patients [10, 13] which is thought to contribute to the pathology of the disease. Although functional CFTR expression is clearly important for airway HCO_3_^−^ secretion, members of the SLC26 family of Cl^−^/HCO_3_^−^ exchangers, SLC26A3, SLC26A4 and SLC26A6, also transport HCO_3_^−^ [57, 61] and previous studies from our laboratory revealed that SLC26A4 (pendrin) participates in HCO_3_^−^ secretion by Calu-3 cells [14]. We proposed that a functional interaction existed between the phosphorylated R domain of CFTR, and the STAS domain of pendrin as described by Ko et al. [25] which enabled CFTR to regulate HCO_3_^−^ secretion via modulation of pendrin-mediated Cl^−^/HCO_3_^−^ exchange [14].

HCO_3_^−^ transport across the serosal membrane is believed to be governed by members of the SLC4 family of transporters, including importers such as the Na^+^/HCO_3_^−^ cotransporters SLC4A4, SLC4A5 and SLC4A7 [11, 26, 54] which act to accumulate HCO_3_^−^ inside the cell. In contrast, SLC4A2, an electroneutral Cl^−^/HCO_3_^−^ anion exchanger (AE2) that exports HCO_3_^−^, plays an important role in the regulation of intracellular pH during cell alkalinization, as well as in the control of cell volume by Cl^−^ uptake [2, 59]. Furthermore, it has been shown that AE2 is expressed in the airways [1, 12, 55] and immunostaining experiments performed on polarized Calu-3 cells have localized the expression of AE2 to the basolateral membrane [32]. Our laboratory [14, 15] and others [24] have recently demonstrated that a functional Cl^−^/HCO_3_^−^ exchanger is present at the basolateral surface of Calu-3 cells under resting conditions, with features consistent with AE2. In addition, Huang et al. [18] showed that intracellular alkalinization produced by basolateral Cl^−^ removal was decreased by 80% in SLC4A2 knockdown Calu-3 cells. We found that this exchanger was almost completely inhibited by elevations in intracellular cAMP [14, 15], which we proposed would enhance cAMP-stimulated transepithelial HCO_3_^−^ secretion, by reducing HCO_3_^−^ export across the basolateral membrane. However, Kim et al. [24] demonstrated that inhibition of CFTR maintained AE2 activity in cAMP-stimulated Calu-3 cells, while Huang et al. [18] suggested that AE2, acting in concert with Na^+^/HCO_3_^−^ cotransporters, was involved in basolateral Cl^−^ loading and HCO_3_^−^ recycling that helped maintain transepithelial Cl^−^ secretion under cAMP-stimulated conditions in Calu-3 cells. Thus, the role of AE2 in airway HCO_3_^−^ and fluid secretion is still controversial and not fully understood. Therefore, the major aim of the present study was to further understand the cellular pathways that regulate basolateral Cl^−^/HCO_3_^−^ exchange activity in human airway epithelial cells to help provide a better understanding of its role in airways HCO_3_^−^ secretion. Our results have uncovered a novel regulation of the Cl^−^/HCO_3_^−^ exchanger by Ca^2+^/calmodulin signalling and by the master protein kinase, CK2 (casein kinase 2). Since CK2 has previously been implicated in regulating HCO_3_^−^ transport by CFTR in secretory epithelia [65], our findings strongly suggest that CK2 is a key regulatory component of transepithelial HCO_3_^−^ transport in the human airways.

## Methods

### Calu-3 cell culture

The human adenocarcinoma-derived cell line, Calu-3 (ATCC HTB-55), was grown in T_75_ Costar cell culture flasks (75 cm^2^) in 30 ml of Eagle’s Minimum Essential Medium (EMEM) supplemented with 10% foetal bovine serum (FBS), 100 U m1^−1^ penicillin and 100 μg ml^−1^ streptomycin, 1% non-essential amino acids, 2 mM L-Glutamine (Sigma) and incubated at 37 °C in a humidified air containing 5% (*v*/*v*) CO_2_. Cells were initially seeded at 3 × 10^6^ cells per flask and passaged every 7 days using 0.05% trypsin and 0.02% ethylenediaminetetraacetic acid (EDTA) in Earle’s balanced salt solution. For intracellular pH measurements, cells were seeded at 250,000 cells cm^−2^ initial seeding density onto semi-permeable Transwell supports (0.4 μm pore, polyester membrane insert, 1.12 cm^2^ surface area, (Corning, UK)). Calu-3 cells generally produced a polarized monolayer after 7 days. All experiments were carried out 8–14 days after seeding.

### Human embryonic kidney-293T (HEK-293T) cell culture

HEK-293T cells were grown in T_75_ Costar cell culture flasks (75 cm^2^) with 30 ml of Dulbecco’s Modified Eagle’s Medium (DMEM), supplemented as for Calu-3 cells and incubated at 37 °C in a humidified air containing 5% (*v*/*v*) CO_2_. Cells were initially seeded at 1 × 10^6^ cells per flask and passaged every 7 days using 0.05% trypsin and 0.02% ethylenediaminetetraacetic acid (EDTA) in Earle’s balanced salt solution. The knockout CK2 (both the alpha (α) and alpha prime (α prime) catalytic subunits) HEK-293T cells were generated by CRISPR/Cas9 gene editing. Genomic target sequences of guide RNA (gRNA) were 5′-TTACATGTATGAGATTCTGA-3′ (CK2 α) and 5′-GGGTCTACGCCGAGGTGAAC-3′ (CK2 α prime). The absence of the catalytic subunits was confirmed by Western blotting (see supplementary Fig. 2). For transient transfection, HEK-293T cells were seeded onto 25 mm glass coverslips (VWR) at 100,000 cells per coverslip and then transiently transfected with cDNA coding for mAE2 or the various CK2 constructs 1 day post-seeding. Intracellular pH measurements were performed 48 h post-transfection.

### Isolation and culture of primary human nasal epithelia

Ethical approval for collection of paediatric human nasal epithelial cells was granted by the NRES Committee North East - Newcastle and North Tyneside 1 (REC reference: 15/NE/0215) and informed written parental consent was obtained. Human nasal epithelial cells were obtained by brushing the inferior nasal turbinate of each nostril from two donors with a cytology brush (Conmed, UK). Nasal epithelial cells were expanded in purified bovine collagen (PureCol; Advanced Biomatrix)-coated T_25_ cell culture flasks (25cm^2^) containing bronchial epithelial growth medium supplemented with SingleQuots (Lonza, Basel, Switzerland), penicillin/streptomycin (100 Units ml^−1^ penicillin and 0.1 mg ml^−1^ streptomycin) and Primocin (InvivoGen, UK). For air liquid interface (ALI) cultures, cells were passaged at 80–90% confluency using 0.05% trypsin and 0.02% EDTA and seeded onto purified collagen coated (PureCol; Advanced Biomatrix) semi-permeable Snapwell supports (0.4 μm pore, polyester membrane insert, 1.12 cm^2^ surface area; Corning, UK) and grown until confluent. The apical medium was subsequently removed and cell cultures were fed basolaterally on alternate days. Cultures were incubated at 37 °C in humidified air containing 5% CO_2_. ALI culture differentiation was characterized through the demonstration of mucus production, cilia formation and serial transepithelial resistance (TEER) measurements (STX2 electrodes, EVOM2™ Epithelial Voltohmmeter, World Precision Instruments) to assess tight junction integrity. Experiments were performed on polarized epithelia after 28 days of ALI culture.

### CK2 kinase activity assay

CK2 activity in cell lysates was measured by means of radioactive assays with [γ-33P]ATP towards the specific CK2 substrate peptide CK2-tide (RRRADDSDDDDD). Briefly, both Calu-3 and HEK-293T cells were treated for 5 min with 10 μM of the indicated compound and then lysed in HEPES 50 mM pH 8, NaCl 150 mM, Glycerol 1% (*w*/*v*), Triton X-100 1% (*v*/*v*), MgCl_2_ 45 mM, EGTA 5 mM with a cocktail of anti-proteases and anti-phosphatases. Protein concentration was determined by the Bradford method. CK2-tide peptide (250 μM) were phosphorylated by incubation at 37 °C for 20 min in a 25-μl volume containing 50 mM Tris/HCl (pH 7.5), 10 mM MgCl_2_, 100 mM NaCl and 100 μM [γ-33P]ATP (specific radioactivity 1000–1500 c.p.m./pmol). The reaction was started by the addition of 1 or 2 μg of lysate and stopped by ice cooling and absorption on phosphocellulose P81 paper. Papers were washed three times with 75 mM phosphoric acid, dried and counted in a scintillation counter.

### Transfection of HEK-293T cells

Mouse AE2 cDNA was a kind gift from Beth Lee and Ron Kopito, and contained a human haemagglutinin (HA) tag; for more details, see Lee et al. [28]. Empty plasmid (pcDNA 3.1 myc/His), human WT-CK2 (CK2-alpha) and the double CK2 mutant (V66A and I174A; DM-CK2) cDNA were generated as previously described [50, 51]. DNA sequencing analysis confirmed sequence identity of all the constructs. To transfect cDNA constructs into HEK-293T cells, cDNA was pre-complexed with Lipofectamine-2000 (Thermo Fisher) at a ratio of 1:2.28, respectively. Opti-MEM media with GlutaMax (Thermo Fisher) was then added for 15 min at room temperature, and then diluted in culture media to produce a final concentration of 1 μg DNA ml^−1^. This complex media was added to the cells and incubated for 6 h at 37 °C before the complex media was removed and cells were incubated with Opti-MEM plus 10% FBS overnight before being returned back to normal culture media.

### Intracellular pH measurements

For measurements in polarized Calu-3 cells and well-differentiated human nasal epithelia, cells were loaded with 10 or 40 μM, respectively, of the pH sensitive dye, 2′-7′-bis (carboxyethyl)-5(6)-carboxyfluorescein acetoxymethyl ester (BCECF-AM) in Na-HEPES and incubated for 60 min at 37 °C. Cells were mounted on to the stage of a Nikon fluor inverted microscope and viewed at ×60 magnification using a long working distance objective (N.A 0.6). Cells were perfused with Krebs solution at 37 °C gassed with 5% (*v*/*v*) CO_2_/95% (*v*/v) O_2_ at a rate of 3 ml min^−1^ (apical) and 6 ml min^−1^ (basolateral). For measurements in HEK-293T cells, cells were loaded with 10 μM BCECF-AM in Na-HEPES and incubated for 10 min at 37 °C. After dye loading, coverslips were placed in a perfusion chamber and then mounted onto the stage of a Nikon inverted microscope. Cells were viewed at ×40 magnification using an oil immersion objective (N.A 1.2) and perfused with Krebs solution at 37 °C gassed with 5% CO_2_/95% O_2_ at a rate of 3 ml min^−1^. Intracellular pH (pH_i_) was measured using a Life Sciences Microfluorimeter System in which cells were alternatively excited at 490 and 440 nm wavelengths every 1.024 s with emitted light collected at 510 nm. The ratio of 490 nm emission to 440 nm emission was recorded using the PhoCal 1.6b software and calibrated to pH_i_ using the high K^+^/nigericin technique [16] in which cells were exposed to high K^+^ solutions containing 10 μM nigericin, set to a desired pH, ranging from 6.6 to 8.4. Total buffering capacity (β_tot_) was calculated by addition of the intrinsic buffering capacity (β_i_) to the buffering capacity of the CO_2_-HCO_3_^−^ buffer system (βHCO_3_^−^) in which β_i_ was calculated using the NH_4_^+^ technique as described by Roos, Boron [49]. For analysis of pH_i_ measurements, delta pH_i_ (ΔpH_i_) was determined by calculating the mean pH_i_ over 60 s before, during and after treatment. Rate of pH_i_ change (ΔpH_i_/Δt) was determined by performing a linear regression over a period of at least 30 s which was converted to a transmembrane HCO_3_^−^ flux (−J(B)) by multiplying ΔpH_i_/Δt by β_tot_.

### Confocal microscopy to detect expression of HA-tagged mAE2

Control, untransfected and transfected HEK-293T cells expressing HA-tagged mAE2 were grown on glass coverslips for 2 days and fixed with 4% PFA for 10 min at room temperature. Cells were washed with PBS three times for 5 min, and then with 50 mM NH_4_Cl to quench any remaining PFA. After washing, fixed cells were permeabilized using 1% Triton X-100 for 5 min at room temperature and washed in PBS three times for 5 min. To block non-specific binding, cells were incubated with blocking buffer, consisting of 5% goat serum and 1% Na-azide in PBS, at room temperature for 30 min. Blocking buffer was removed and cells were incubated in diluted primary antibody (Anti-HA16B12, 1/1000 in blocking buffer, Abcam) overnight at 4 °C on a shaker. Cells were then rinsed in PBS three times for 15 min to remove any unbound primary antibody, and then incubated with FITC-conjugated goat anti-mouse antibody (1/100 in blocking buffer) for 1 h at room temperature in the dark. Following this, cells were washed with PBS three times for 15 min to remove any unbounded secondary antibody. DAPI dye (1 μg ml^−1^) was added onto coverslips for 2 min, at room temperature, away from light, to stain the nucleus and gently washed in PBS to remove any remaining DAPI. Coverslips were mounted onto a microscope slide, using mounting medium (VectaShield). Cells were observed under Nikon A1R Confocal microscope at ×60 magnification (0.1 DIC lens) with a numerical aperture of 1.4. Cells were excited with the DAPI excitation wavelength of 405 nm to visualize DAPI-stained specimens, and imaged at the emission wavelength of 450 nm. To visualize FITC-stained specimens, cells were excited with the FITC-excitation wavelength of 495 nm and imaged at the emission wavelength of 517 nm.

### Solutions and reagents

All reagents and inhibitors were purchased from Sigma-Aldrich (Sigma-Aldrich Company Ltd., UK), apart from forskolin, BAPTA-AM and TBB (R & D Systems); BCECF-AM, DIDS, Lipofectamine 2000, WGA (Thermo Fisher), GlyH-101 and J-8 (Santa Cruz), RpcAMPs (Enzo life science), and Anti-HA antibody (Abcam). CX-4945 was produced by Glixx Laboratories (Glixx Laboratories, MA, USA). All stock solutions of agonists and inhibitors were made in DMSO, apart from nigericin (made in 100% ethanol), and 8CPT-2Me-cAMP, carbachol and adenosine (dissolved in deionized water). gRNAs designs and reagents for genome editing were supplied by Horizon Discovery (www.horizondiscovery.com). The HCO_3_^−^ free Na-HEPES buffer solution consisted of (in mM) 130 NaCl, 5 KCl, 1 CaCl_2_, 1 MgCl_2_, 10 Na-HEPES and 10 D-Glucose. The Cl^−^-free HEPES buffer solution consisted of (in mM) 130 Na-gluconate, 2.5 K_2_SO_4_, 6 Ca-gluconate, 1 Mg-gluconate, 10 HEPES (free acid) and 10 D-Glucose. All HEPES-buffered solutions were calibrated to pH 7.4 by addition of 1 M HCl. High Cl^−^- Krebs solution consisted of (in mM) 25 NaHCO_3_^−^, 115 NaCl, 5 KCl, 1 CaCl_2_, 1 MgCl_2_ and 10 D-Glucose. In the Ca^2+^-free high Cl^−^- Krebs solution, the NaCl concentration was increased to 116 mM, and CaCl_2_ was replaced with MgCl_2_ and 0.5 mM EGTA was added to chelate any remaining Ca^2+^. The Cl^−^-free Krebs solution consisted of (in mM) 25 NaHCO_3_^−^, 115 Na-Gluconate, 2.5 K_2_SO_4_, 6 Ca-gluconate, 1 Mg gluconate and 10 D-Glucose. In the Ca^2+^-free Cl^−^-free Krebs solution, the Na-gluconate concentration was increased to 124 mM, and 0.5 mM EGTA was added to chelate any remaining Ca^2+^. The intracellular pH calibration solutions consisted of (in mM) 5 NaCl, 130 KCl, 1 CaCl_2_, 1MgCl_2_, 10 D-Glucose, 10 HEPES (for solutions set at pH 7.6 or below) or 10 TRIS (for solutions set at pH 7.8 or above) as well as 10 μM nigericin. Solutions were set to the desired pH by using either 1 M HCl or 1 M NaOH. The ammonium pulse solutions used to determine intracellular buffering capacity consisted of (in mM) 4.5 KCl, 1MgCl_2_, 2 CaCl_2_, 5 BaCl, 10 HEPES, 10 D-Glucose as well as varying concentrations of NH_4_Cl/NMDG-Cl, ranging from 0 NH_4_Cl/145 NMDG-Cl to 30 NH_4_ Cl/115 NMDG-Cl. All solutions were titrated to pH 7.4 at 37 °C using 1 M CsOH.

### Statistical analysis

All results are presented as mean ± S.E.M. where *n* is the number of experiments. The GraphPad Prism 4 software (GraphPad Software, USA) was used for statistical analysis and either a Student’s *t* test (paired or unpaired), one-way ANOVA (with Tukey’s multiple comparison post-test) or two-way ANOVA (with Bonferroni’s post-test), where applicable. *p* values of <0.05 were considered statistically significant.

## Results

### Calu-3 cells express a basolateral DIDS-sensitive, Cl^−^/HCO_3_^−^ exchanger

**Fig. 1:**
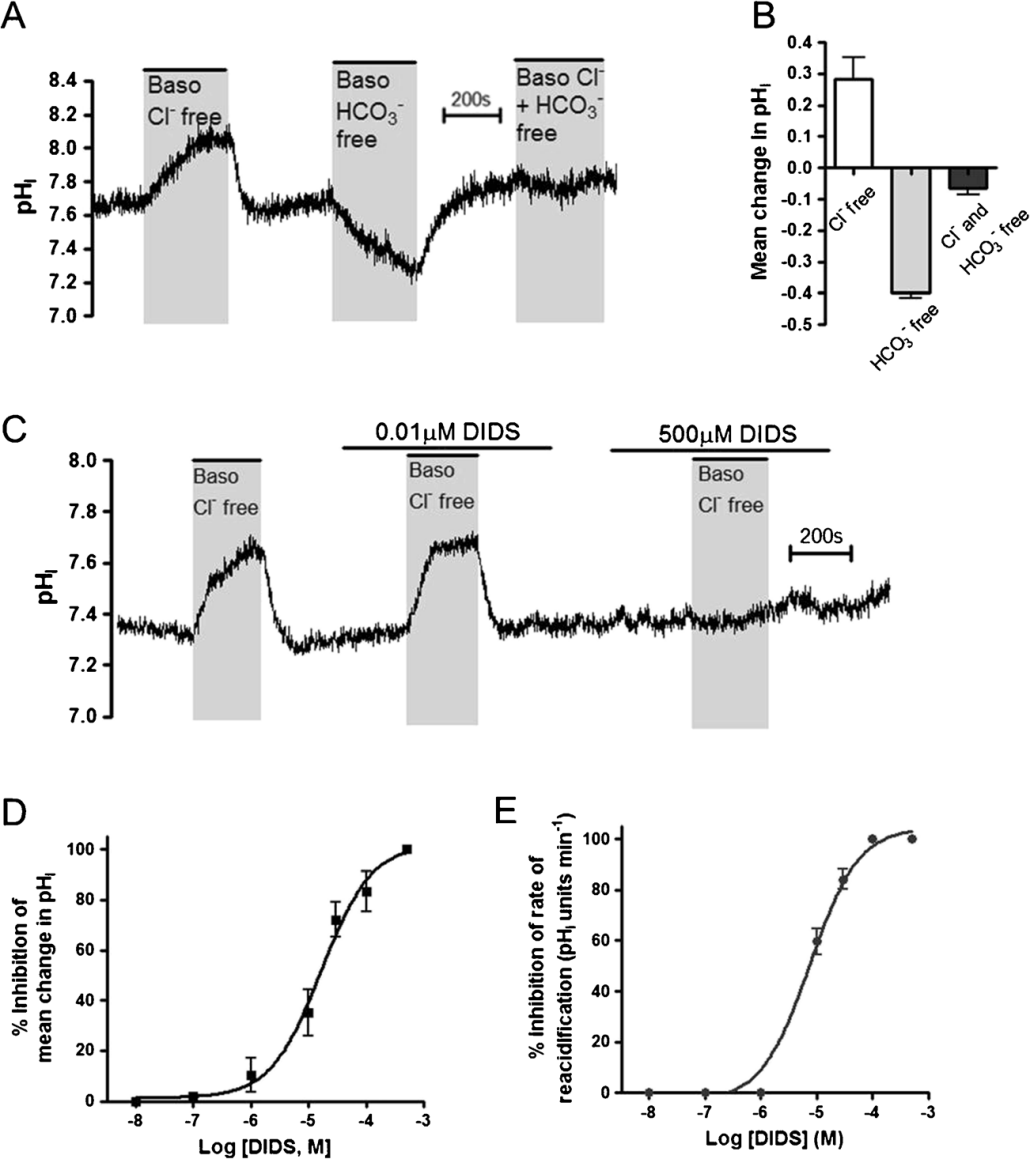
Calu-3 cells express a DIDS-sensitive Cl^−^ and HCO_3_^−^ dependent anion exchanger on the basolateral membrane: Calu-3 cells were loaded with BCECF-AM (10 μM; 1 h) and pH_i_ was determined by fluorescent microscopy. **a** Shows an example experiment in which basolateral Cl^−^ was removed and replaced by gluconate, basolateral HCO_3_- was removed and replaced by HEPES or both Cl^−^ and HCO_3_^−^ were removed, as indicated. **b** Summarizes the mean change in pH_i_ resulting from anion replacement. Data represents mean ± S.E.M., *n* = 3–6. **c** Shows an example experiment in which the activity of the basolateral Cl^−^/HCO_3_^−^ exchanger was assessed by measuring pH_i_ changes in response to replacement of basolateral Cl^−^ with gluconate in the presence of DIDS (0.01 or 500 μM). **d** Displays the percent inhibition of the mean change in pH_i_ caused by basolateral Cl^−^ removal and **e** the percent inhibition of the rate of reacidification upon Cl^−^ readdition at different DIDS concentrations. Note that the data points of DIDS concentration of 10^−8^, 10^−7^ and 10^−6^ M are plotted but lie directly on the *x* axis. In each case, a non-linear regression was fit to the data. Data represents mean ± S.E.M. (*n* = 3–8)

Our laboratory [14, 15] and others [24] have previously reported that Cl^−^/HCO_3_^−^ exchange occurs across the basolateral membrane in non-stimulated Calu-3 cells. In support of these findings, intracellular pH measurements showed that removal of basolateral Cl^−^ caused an intracellular alkalinization of 0.36 ± 0.02 units (*n* = 8; Fig. 1A) and that readdition of Cl^−^ caused pH_i_ to reacidify at a rate of 0.57 ± 0.07 pH units min^−1^ (*n* = 8). Similarly, removal of basolateral HCO_3_^−^, and replacing it with HEPES, induced an intracellular acidification, indicating enhanced basolateral HCO_3_^−^ efflux down its concentration gradient. However, there was no measurable pH change in response to removal of basolateral Cl^−^ in the absence of basolateral HCO_3_^−^ (Fig. 1A, B). Overall, these data are consistent with Cl^−^/HCO_3_^−^ exchange occurring across the basolateral membrane. The anion exchange inhibitor 4,4′-Diisothiocyano-2,2′-stilbenedisulfonic acid (DIDS) dose-dependently inhibited this basolateral Cl^−^/HCO_3_^−^ exchange activity, with 500 μM DIDS causing complete inhibition (Fig. 1C). Analysis of the DIDS dose-response curves for inhibition of the change in pH_i_ following basolateral Cl^−^ removal, and rate of reacidification following basolateral Cl^−^ reintroduction, gave a calculated IC_50_ value of 16.5 ± 1.3 μM and 7.5 ± 1.2 μM, respectively (Fig. 1D, E). Therefore, these data demonstrate that Calu-3 cells have a DIDS-sensitive, basolateral Cl^−^/HCO_3_^−^ anion exchanger with properties consistent with SLC4A2 (AE2), which support previous results from these cells [14, 15, 24].

### The basolateral Cl^−^/HCO_3_^−^ exchanger is inhibited by elevations in cytosolic cAMP

**Fig. 2:**
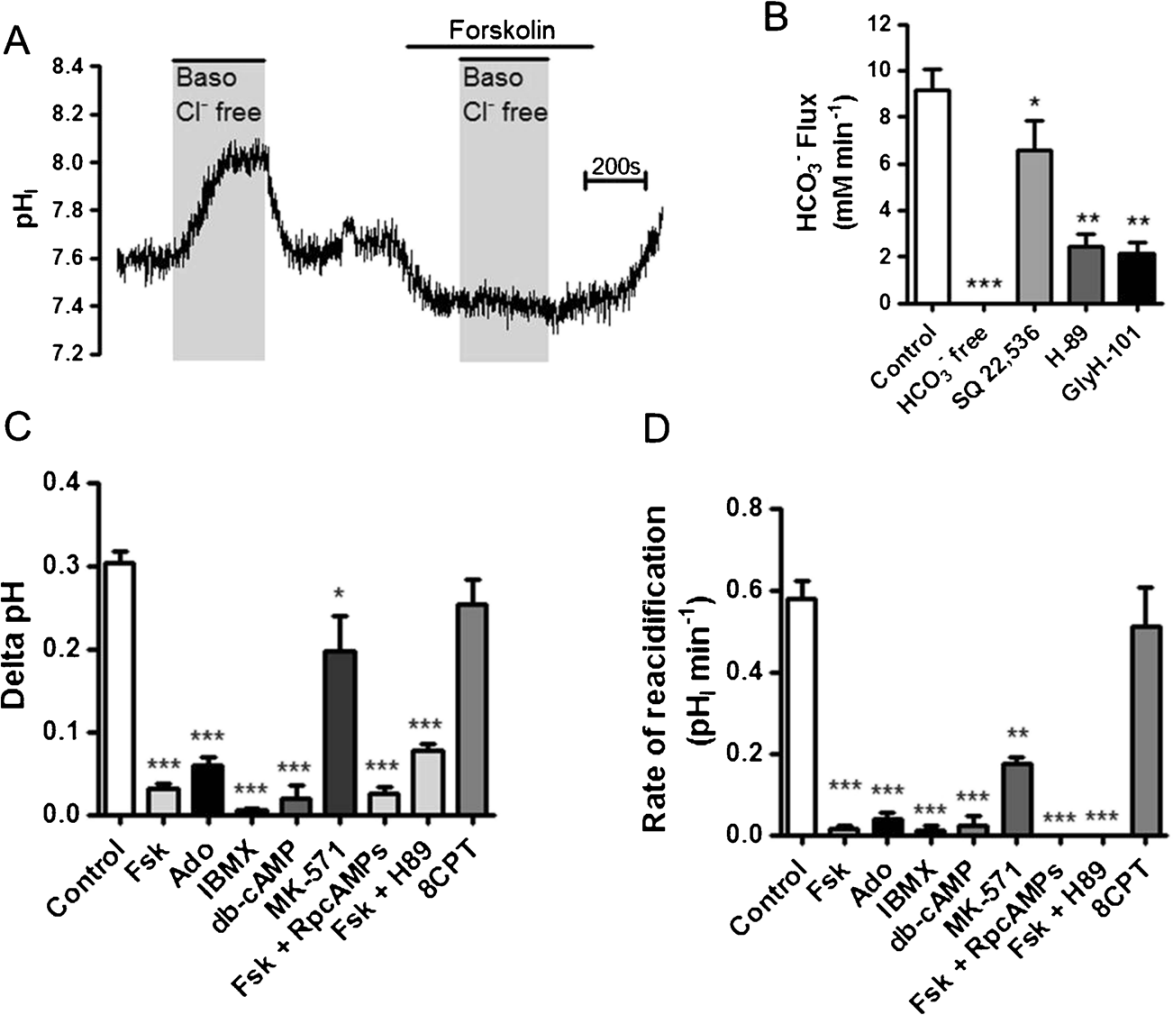
The basolateral Cl^−^/HCO_3_^−^ exchanger is negatively regulated by cAMP: Calu-3 cells were loaded with BCECF-AM (10 μM; 1 h) and pH_i_ was determined by fluorescent microscopy. The activity of the basolateral Cl^−^/HCO_3_^−^ exchanger was assessed by measuring pH_i_ changes in response to replacement of basolateral Cl^−^ with gluconate. **a** Shows a representative experiment in which the activity of the Cl^−^/HCO_3_^−^ exchanger was assessed in the presence of forskolin (5 μM). **b** Summarizes the forskolin-stimulated HCO_3_^−^ efflux in control cells or in conditions in which HCO_3_^−^ was removed and replaced by HEPES, or in cells pretreated with the transmembrane adenylyl cyclase inhibitor SQ 22,536 (500 μM), the PKA inhibitor H-89 (50 μM) or the CFTR inhibitor GlyH-101 (10 μM) *Significant difference vs. control (*p* < 0.05; ** = *p* < 0.01; *** = *p* < 0.001). Data represents mean ± S.E.M., *n* = 3–22. **c** and **d** summarize the effect of forskolin (Fsk; 5 μM), adenosine (Ado; 10 μM), IBMX (1 mM), dibutryl-cAMP (db-cAMP; 800 μM), MK-571 (10 μM), Fsk in cells pretreated with RpcAMPs (1 mM; 1 h), Fsk in cells pretreated with H89 (50 μM; 1 h) and the EPAC agonist 8CPT-2Me-cAMP-AM (10 μM; 1 h) on the mean change in pH_i_ caused by basolateral Cl^−^ removal and the rate of reacidification upon Cl^−^ readdition, respectively. *Significant effect of treatment vs. control (*p* < 0.05; ** = *p* < 0.01; *** = *p* < 0.001). Data represents mean ± S.E.M., *n* = 3–30

The next series of experiments were designed to investigate the regulation of the basolateral Cl^−^/HCO_3_^−^ exchanger. We first sought to assess the effect of elevated cAMP on Cl^−^/HCO_3_^−^ exchanger activity, since cAMP is known to stimulate transepithelial HCO_3_^−^ secretion from Calu-3 cells in a PKA-dependent manner [14]. As shown in Fig. 2A, addition of forskolin to Calu-3 cells induced an intracellular acidification of 0.17 ± 0.01 pH_i_ units (*n* = 22), which gave a calculated HCO_3_^−^ efflux of 9.2 ± 0.9 mM min^−1^ (*n* = 22). This forskolin-stimulated acidification was absent when HCO_3_^−^ was replaced by HEPES and was sensitive to the transmembrane adenylyl cyclase inhibitor SQ 22,536, the PKA inhibitor H-89 and the CFTR inhibitor GlyH-101 (Fig. 2B), indicating that this response to forskolin was a result of cAMP-stimulated, CFTR-dependent HCO_3_^−^ secretion. Importantly, although forskolin promoted HCO_3_^−^ efflux across the apical membrane, it appeared to markedly reduce HCO_3_^−^ efflux across the basolateral membrane by the anion exchanger (Fig. 2). Forskolin reduced the mean change in pH_i_ following basolateral Cl^−^ removal by 85.2 ± 2.6% compared to non-stimulated cells (*p* < 0.001 vs. control; *n* = 10; Fig. 2C) and also reduced the rate of reacidification following basolateral Cl^−^ reintroduction by 98.4 ± 1.6% compared to non-stimulated cells (*p* < 0.001 vs. control; *n* = 10; Fig. 2D). Similar inhibition of the basolateral Cl^−^/HCO_3_^−^ exchanger was also observed in cells stimulated with the cAMP-elevating agonist adenosine (Fig. 2C), a key physiological regulator of cAMP-stimulated ion and fluid transport in human airways [48, 63]. In addition, the non-specific phosphodiesterase inhibitor IBMX, and the membrane permeable cAMP analogue dibutryl-cAMP (db-cAMP), both induced almost complete inhibition of the basolateral Cl^−^/HCO_3_^−^ exchanger (Fig. 2C, D). The multidrug resistance protein 4 (MRP4) inhibitor MK-571 also induced an inhibition of the exchanger, but this was less pronounced than for other cAMP agonists (Fig. 2C, D). Therefore, elevations of [cAMP]_i_ via different mechanisms, i.e. (i) activation of adenylyl cyclase (forskolin and adenosine), (ii) inhibition of cAMP breakdown (IBMX) or (iii) inhibition of MRP4-dependent cAMP efflux (MK-571) [67] demonstrated that the basolateral Cl^−^/HCO_3_^−^ exchanger was negatively regulated by cAMP. However, inhibition of PKA by two different inhibitors, RpcAMPs and H89, had no effect on the ability of forskolin to reduce AE activity (Fig. 2C, D). Note that this result is in marked contrast to the effect these PKA inhibitors have on the forskolin-activated, CFTR-dependent, apical anion exchanger pendrin, which was reduced by over 80% by H89 [14]. In addition, we also found that the exchange protein activated by cAMP (EPAC) and mTOR, two reported downstream targets of cAMP which are activated independently of PKA [23, 52], were not involved in mediating the effect of cAMP on the basolateral Cl^−^/HCO_3_^−^ exchanger (Fig. 2C, D). Activation of EPAC by 8CPT-2Me-cAMP-AM failed to mimic the effect of forskolin, with similar Cl^−^/HCO_3_^−^ exchanger activity compared to control cells (*n* = 3; Fig. 2C, D). Inhibition of mTOR kinase by rapamycin, also had no effect on the forskolin-induced inhibition of exchanger (*n* = 3: data not shown).

**Fig. 3:**
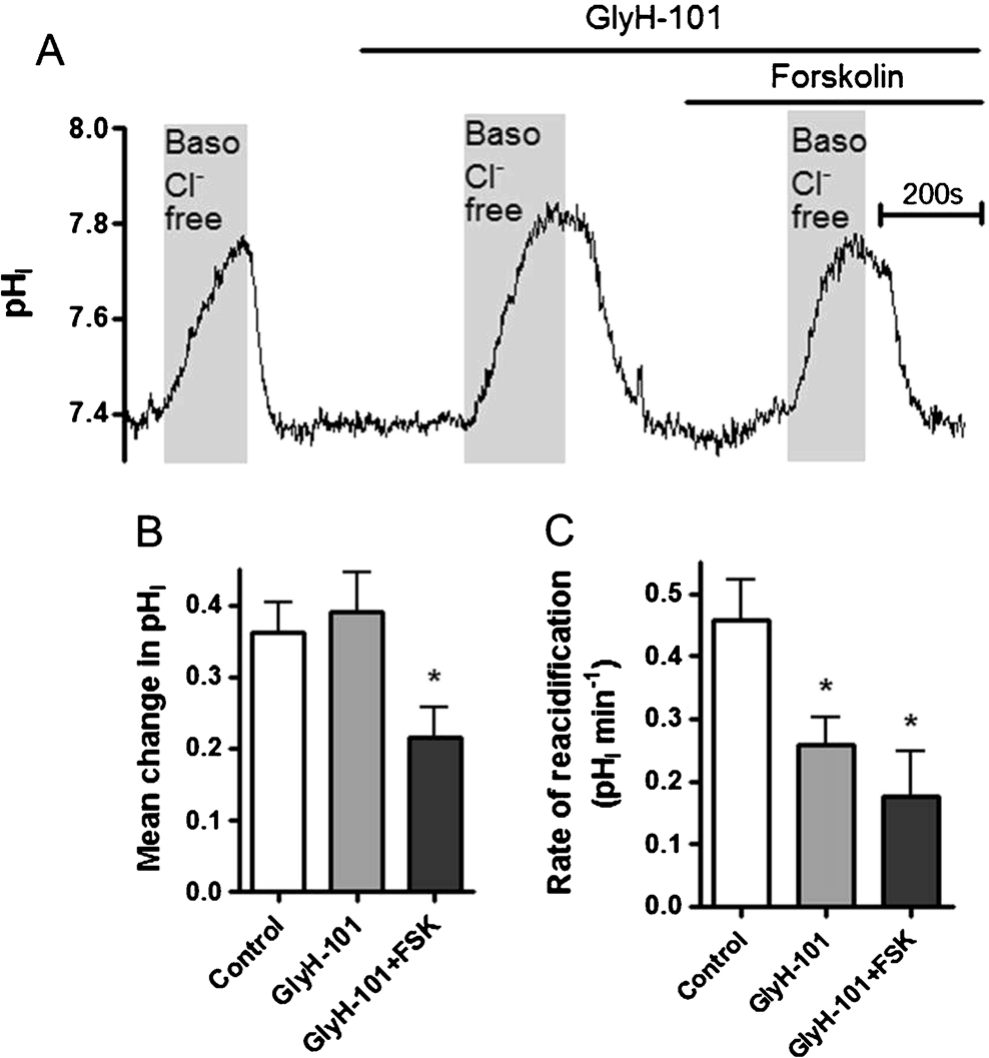
GlyH-101 partially, but not fully, relieves forskolin-induced inhibition of the basolateral Cl^−^/HCO_3_^−^ exchanger: Calu-3 cells were loaded with BCECF-AM (10 μM; 1 h) and pH_i_ was determined by fluorescent microscopy. The activity of the basolateral Cl^−^/HCO_3_^−^ exchanger was assessed by measuring pH_i_ changes in response to replacement of basolateral Cl^−^ with gluconate. **a** Shows a representative experiment in which exchanger activity was assessed under basal conditions and in the presence of GlyH-101 (10 μM) and forskolin (5 μM). **b** and **c** summarize the effect of GlyH-101 alone and in the presence of forskolin on the mean change in pH_i_ caused by basolateral Cl^−^ removal and the rate of reacidification upon Cl^−^ readdition, respectively. *Significant effect of treatment vs. control (*p* < 0.05). Data represents mean ± S.E.M., *n* = 4

In another series of experiments, we tested whether forskolin-stimulated HCO_3_^−^ secretion at the apical membrane could potentially mask any basolateral Cl^−^/HCO_3_^−^ exchange activity, as suggested by Kim et al. [24]. Addition of the CFTR inhibitor, GlyH-101, partially, but not fully, relieved the forskolin-induced inhibition of the basolateral Cl^−^/HCO_3_^−^ exchanger (Fig. 3). Given that the percent inhibition of the rate of reacidification in response to reintroduction of basolateral Cl^−^ was 65.2 ± 14.3% (*n* = 4; Fig. 3C) in the presence of apical GlyH-101 compared to 97.3 ± 1.3% in control (+FSK) conditions (*n* = 16; Fig. 2D; *p* < 0.001), suggested that apical HCO_3_^−^ secretion under forskolin-stimulated conditions masked some pH_i_ changes resulting from basolateral HCO_3_^−^ influx via AE2 in response to removal of basolateral Cl^−^. However, because there was still a significant decrease of basolateral Cl^−^/HCO_3_^−^ exchanger activity by forskolin, even in the presence of GlyH-101, demonstrates that AE2 is negatively regulated by increases in cAMP. However, this effect appears to be independent of PKA. Thus, the mechanism involved in regulation of the basolateral Cl^−^/HCO_3_^−^ exchanger in Calu-3 cells by cAMP still needs to be resolved.

### The basolateral Cl^−^/HCO_3_^−^ exchanger is regulated by resting levels of Ca^2+^ and calmodulin signalling

**Fig. 4:**
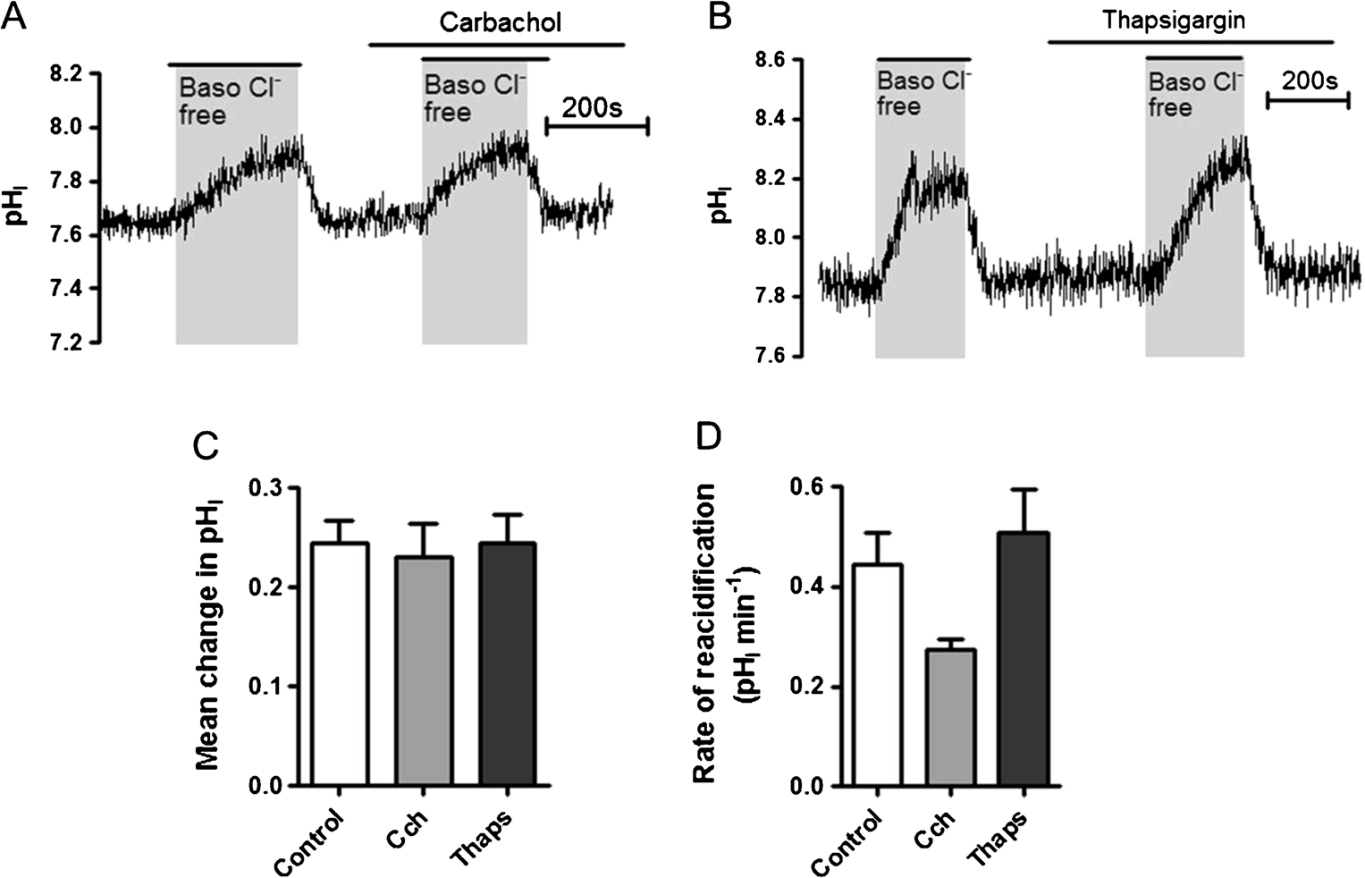
Elevations in intracellular Ca^2+^ have no effect on the activity of the basolateral Cl^−^/HCO_3_^−^ anion exchanger: Calu-3 cells were loaded with BCECF-AM (10 μM; 1 h) and pH_i_ was determined by fluorescent microscopy. The activity of the basolateral Cl^−^/HCO_3_^−^ exchanger was assessed by measuring pH_i_ changes in response to replacement of basolateral Cl^−^ with gluconate. **a** Shows a representative experiment in which basolateral Cl^−^/HCO_3_^−^ anion exchanger activity was assessed in the presence of basolateral carbachol (Cch; 20 μM) and **b** shows a representative experiment in which basolateral Cl^−^/HCO_3_^−^ anion exchanger activity was assessed in the presence of thapsigargin (Thaps; 200 nM). **c** and **d** summarize the effect of carbachol and thapsigargin stimulation on the mean change in pH_i_ caused by basolateral Cl^−^ removal and the rate of reacidification upon Cl^−^ readdition, respectively. Data represents mean ± S.E.M., *n* = 3–13

Having shown that the ubiquitous second messenger cAMP played an important role in the regulation of the basolateral Cl^−^/HCO_3_^−^ exchanger in Calu-3 cells, we next investigated its regulation by Ca^2+^, another key second messenger. Not only are Ca^2+^ and cAMP signalling generally synergistic in secretory epithelia [9, 27, 38], it has also been demonstrated that the basolateral Cl^−^/HCO_3_^−^ exchanger in mouse salivary acinar cells is positively regulated by intracellular Ca^2+^ [39]. To this end, we first tested whether elevations in intracellular Ca^2+^ affected the activity of the basolateral Cl^−^/HCO_3_^−^ exchanger. Therefore, Calu-3 cells were stimulated with either basolateral carbachol, a muscarinic receptor agonist, (Fig. 4A), or thapsigargin, an inhibitor of sarcoplasmic/endoplasmic reticulum Ca^2+^ ATPase (SERCA), (Fig. 4B), which have both been shown to stimulate Ca^2+^-dependent anion transport in Calu-3 cells [37]. However, as shown in Fig. 4C, D, neither carbachol nor thapsigargin had any significant effect on basolateral Cl^−^/HCO_3_^−^ exchanger activity. Therefore, these data suggest that the exchanger is not regulated by [Ca^2+^]_i_.

**Fig. 5:**
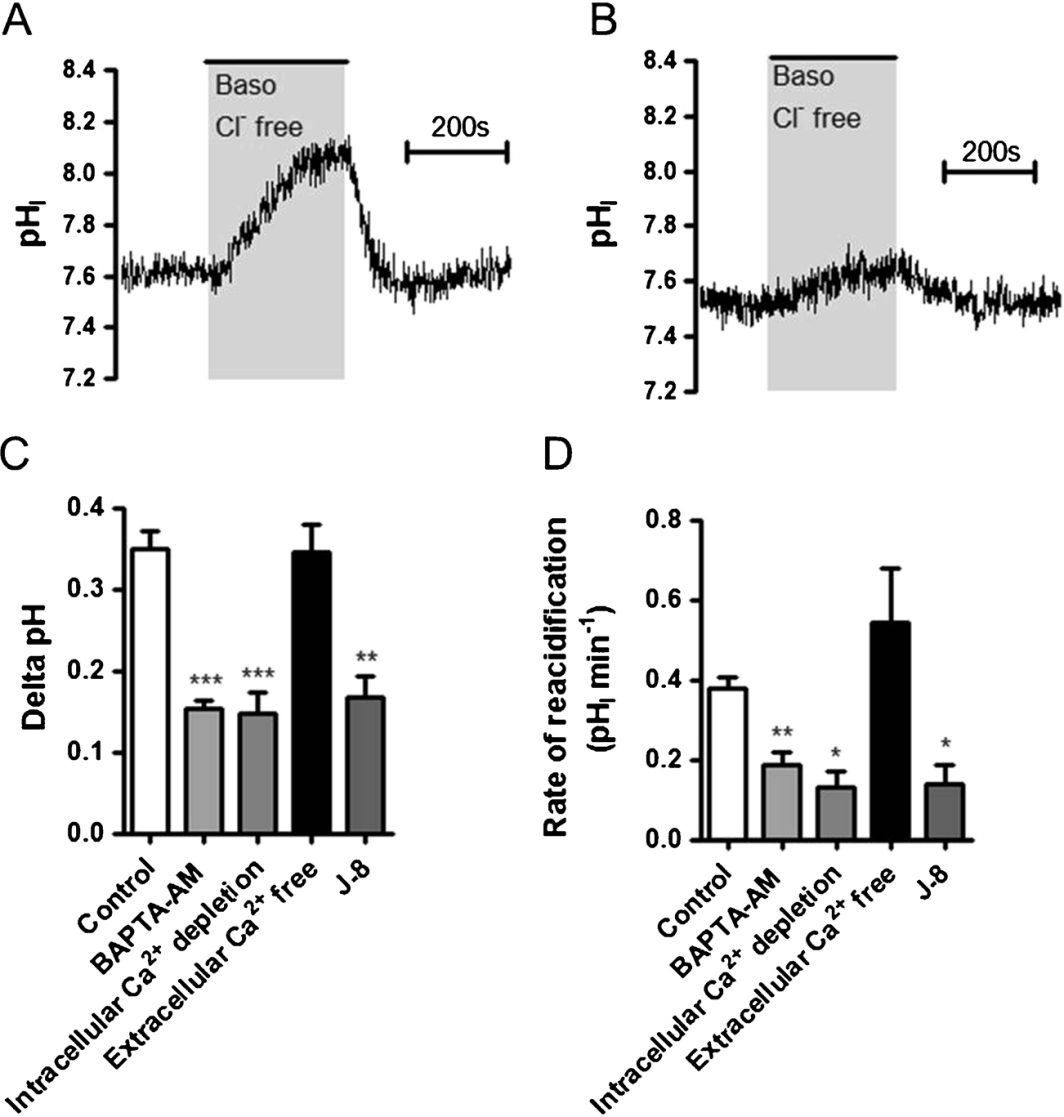
Intracellular Ca^2+^ and calmodulin signalling regulate the basolateral Cl^−^/HCO_3_^−^ exchanger: Calu-3 cells were loaded with BCECF-AM (10 μM; 1 h) and pH_i_ was determined by fluorescent microscopy. The activity of the basolateral Cl^−^/HCO_3_^−^ exchanger was assessed by measuring pH_i_ changes in response to replacement of basolateral Cl^−^ with gluconate. **a** Shows a representative control experiment and **b** shows an experiment in which cells had been preincubated with BAPTA-AM (50 μM) for 1 h prior to study. **c** and **d** summarize the effect of BAPTA-AM (50 μM), depletion of intracellular Ca^2+^ stores via stimulation with thapsigargin (200 nM) in the absence of extracellular Ca^2+^, removal of extracellular Ca^2+^, and J-8 (50 μM), on the mean change in pH_i_ caused by basolateral Cl^−^ removal and the rate of reacidification upon Cl^−^ readdition, respectively. *Significant effect of treatment vs. control (*p* < 0.05; ** = *p* < 0.01; *** = *p* < 0.001). Data represents mean ± S.E.M., *n* = 3–19

We next tested whether basal levels of [Ca^2+^]_i_ had any effect on anion exchanger activity. To do this, Calu-3 cells were preloaded with the calcium-chelator, BAPTA-AM, and then basolateral Cl^−^/HCO_3_^−^ exchanger activity assessed (Fig. 5A, B). BAPTA-AM loading did not affect resting pH_i_ (control = 7.60 ± 0.06; *n* = 7 and BAPTA-AM-loaded cells = 7.70 ± 0.02; *n* = 5; *p* > 0.05), but did significantly reduce the mean change in pH_i_ following basolateral Cl^−^ removal by 56.2 ± 2.9% (*p* < 0.001 *n* = 8; Fig. 5C), as well as the rate of reacidification following basolateral Cl^−^ reintroduction, by 51.3 ± 8.9% (*p* < 0.01; *n* = 8; Fig. 5D) compared to control cells. However, BAPTA-AM-loaded cells still showed normal forskolin-induced inhibition of the remaining anion exchange activity. These results suggest that resting levels of intracellular Ca^2+^ play an important role in regulating basolateral Cl^−^/HCO_3_^−^ exchange activity. Consistent with a Ca^2+^-dependent regulation of the basolateral Cl^−^/HCO_3_^−^ exchanger, depletion of intracellular Ca^2+^ stores (by removal of external Ca^2+^ and inducing calcium-store depletion with thapsigargin) caused a marked reduction in basolateral Cl^−^/HCO_3_^−^ exchanger activity, with both the change in pH_i_ following basolateral Cl^−^ removal and the rate of reacidification following Cl^−^ readdition reduced by 57.6 ± 7.4% and 65.1 ± 9.8%, respectively, compared to the control response (*p* < 0.05; *n* = 4; Fig. 5C, D). In contrast, removal of extracellular Ca^2+^ had no effect on Cl^−^/HCO_3_^−^ exchanger activity (Fig. 5C, D), indicating that intracellular Ca^2+^ specifically regulated anion exchange activity. Therefore, these data demonstrate that the basal levels of [Ca^2+^]_i_ are sufficient to fully activate the basolateral Cl^−^/HCO_3_^−^ exchanger such that increasing [Ca^2+^]_i_ does not further modulate exchanger activity.

To gain insights into the mechanism underlying the Ca^2+^-dependent regulation of the basolateral Cl^−^/HCO_3_^−^ exchanger, we investigated the role of calmodulin (CaM), an important downstream calcium-binding protein. Treatment of cells with the highly specific CaM inhibitor N-(8-aminooctyl)-5-iodonaphthalene-1-sulfonamide (J-8) [64] significantly reduced the mean change in pH_i_ following basolateral Cl^−^ removal by 52.1 ± 7.8% compared to untreated control cells (*p* < 0.01; *n* = 3; Fig. 5C) and also reduced the rate of reacidification following basolateral Cl^−^ reintroduction by 64.0 ± 13.6% compared to control cells (*p* < 0.05; *n* = 3; Fig. 5D). Thus, these data further support the finding that the resting level of Ca^2+^ is important in regulating basolateral Cl^−^/HCO_3_^−^ exchanger activity, possibly through Ca^2+^-release from thapsigargin-sensitive intracellular stores and subsequent activation of CaM under basal conditions.

### CK2 plays a major role in the regulation of the basolateral Cl^−^/HCO_3_^−^ exchanger in Calu-3 cells

**Fig. 6:**
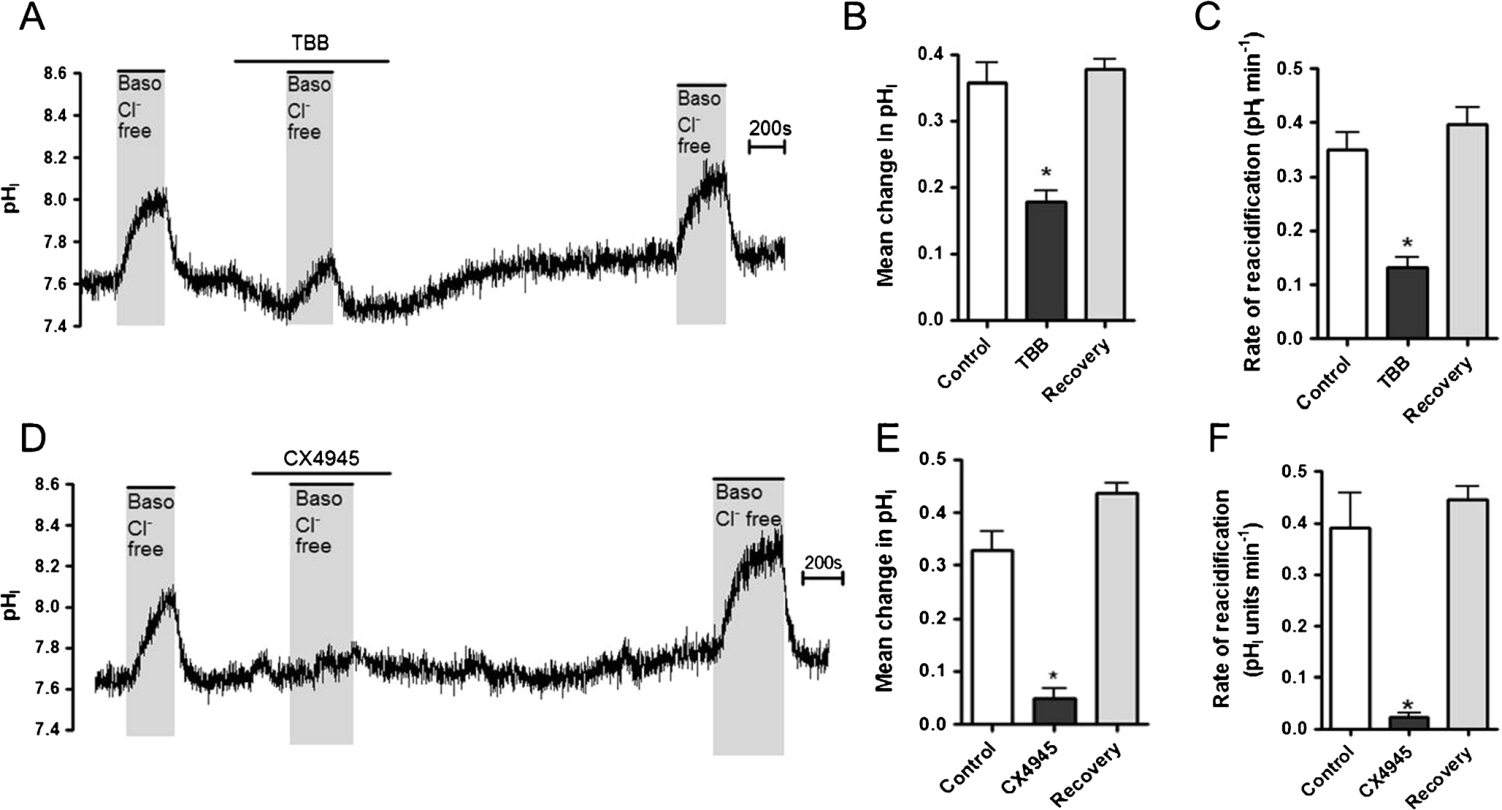
CK2 inhibition by TBB or CX4945 reduces the activity of the basolateral Cl^−^/HCO_3_^−^ exchanger: Calu-3 cells were loaded with BCECF-AM (10 μM; 1 h) and pH_i_ was determined by fluorescent microscopy. The activity of the basolateral Cl^−^/HCO_3_^−^ exchanger was assessed by measuring pH_i_ changes in response to replacement of basolateral Cl^−^ with gluconate. **a** Shows a representative experiment in which Calu-3 cells were exposed to TBB (10 μM) for 5 min and Cl^−^/HCO_3_^−^ exchanger activity measured. **b** and **c** summarize the effect of TBB treatment and TBB reversibility on the mean change in pH_i_ caused by basolateral Cl^−^ removal and the rate of reacidification upon Cl^−^ readdition, respectively. *Significant effect of TBB vs. untreated controls (*p* < 0.05). Data represents mean ± S.E.M., *n* = 6. **d** Shows a representative experiment in which Calu-3 cells were exposed to CX4945 (10 μM) for 5 min and Cl^−^/HCO_3_^−^ exchanger activity measured. **e** and **f** summarize the effect of CX4945 treatment and CX4945 reversibility on the mean change in pHi caused by basolateral Cl^−^ removal and the rate of reacidification upon Cl^−^ readdition, respectively. *Significant effect of CX4945 vs. untreated controls (*p* < 0.05). Data represents mean ± S.E.M., *n* = 6

**Fig. 7:**
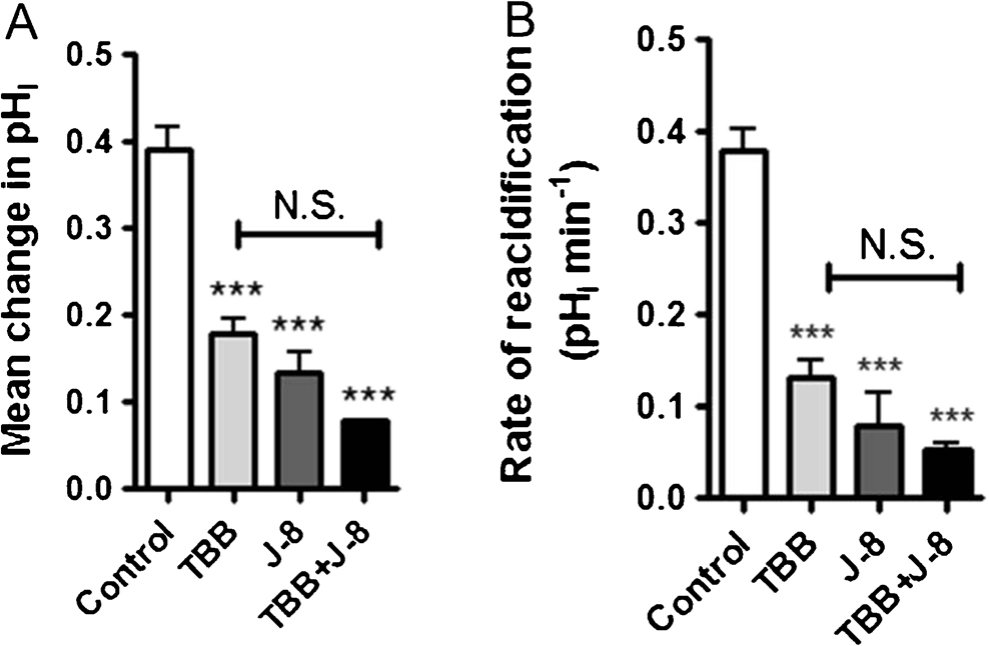
CK2 and CaM regulate the basolateral Cl^−^/HCO_3_^−^ exchanger via similar signalling pathways: Calu-3 cells were loaded with BCECF-AM (10 μM; 1 h) and pH_i_ was determined by fluorescent microscopy. The activity of the basolateral Cl^−^/HCO_3_^−^ exchanger was assessed by measuring pH_i_ changes in response to replacement of basolateral Cl^−^ with gluconate. **a** and **b** display the effect of TBB (10 μM; 5 min exposure), J-8 preincubation (50 μM, 1 h) and J-8 preincubation + acute TBB treatment on the activity of the basolateral Cl^−^/HCO_3_^-^ exchanger, assessed by measuring the mean change in pH_i_ caused by basolateral Cl^−^ removal and the rate of reacidification upon Cl^−^ readdition respectively. ***Significant effect of treatment (*p* < 0.001); *N.S.* non significant (*p* > 0.05). Data represents mean ± S.E.M., *n* = 3–6

In order to further investigate the mechanism behind Ca^2+^/CaM regulation of the basolateral Cl^−^/HCO_3_^−^ exchanger under resting conditions, experiments were performed to assess the role of CK2, a protein kinase that is active under resting conditions in airway cells [65], and which is the main serine/threonine kinase both in vivo and in vitro that can phosphorylate CaM [3]. Furthermore, CK2 has been implicated in the regulation of other ion channels including CFTR [7, 33, 36] and M-type potassium channels, in which it has been reported that CK2-dependent phosphorylation of CaM underlies its binding to the channel [21]. In addition, sequence analysis of SLC4A2 reveals the presence of several potential CK2 phosphorylation sites showing the canonical CK2 consensus sequence pS/pTxxE/D (Supplementary Fig. 1). Calu-3 cells were therefore exposed to the CK2 inhibitor 4,5,6,7-Tetrabromobenzotriazole (TBB) and then basolateral Cl^−^/HCO_3_^−^ exchange activity measured (Fig. 6A). TBB exposure alone caused a small intracellular acidification (7.6 ± 0.02 to 7.5 ± 0.01 (*p* < 0.05, *n* = 6)), and caused a significant, but fully reversible, inhibition of the basolateral Cl^−^/HCO_3_^−^ exchanger (Fig. 6A–C). Since the mean pH_i_ after TBB exposure was within the normal range for Calu-3 cells, it was unlikely that the fall in pH_i_ caused the decrease in basolateral AE activity. However, to provide further support for a role of CK2 in regulating anion exchange activity, the effect of a more specific CK2 inhibitor, CX4945 [45] was tested. Figure 6D shows that exposure to CX4945 almost completely abolished Cl^−^/HCO_3_^−^ exchanger activity, an effect which was fully reversible on washout of the inhibitor, and which did not involve a change in pH_i_ (Fig. 6E, F). The use of two different pharmacological inhibitors provided strong evidence that CK2 was essential for basal Cl^−^/HCO_3_^−^ exchanger activity in Calu-3 cells. The efficacy of a short-term exposure to the two inhibitors on the activity of CK2 was further verified by an in vitro kinase assay of whole cell lysates from Calu-3, as detailed in the ‘Methods’ section. Figure 13A shows that a 5-min treatment of Calu-3 cells with 10 μM TBB lowered CK2 catalytic activity by more than 40%, whereas the same concentration of CX4945 led to an even more dramatic inhibition. In order to investigate whether CK2 regulation of the basolateral Cl^−^/HCO_3_^−^ exchanger involved CaM, Calu-3 cells were preincubated with the CaM inhibitor J-8 for 60 min, and then cells were acutely exposed to TBB, before basolateral Cl^−^/HCO_3_^−^ exchanger activity was measured. Although TBB + J-8 caused a trend to further reduce AE activity compared to either drug alone, this effect was not statistically significant and, even in the presence of both drugs, a significant amount of AE activity still remained (Fig. 7A, B). Since there was no obvious additive effect of TBB and J-8 suggests that CK2 potentially controls the resting activity of the basolateral Cl^−^/HCO_3_^−^ exchanger through the downstream target CaM, in Calu-3 cells.

### CK2 inhibition abolishes the activity of basolateral cl^−^/HCO_3_^−^ exchange in primary human nasal epithelia

**Fig. 8:**
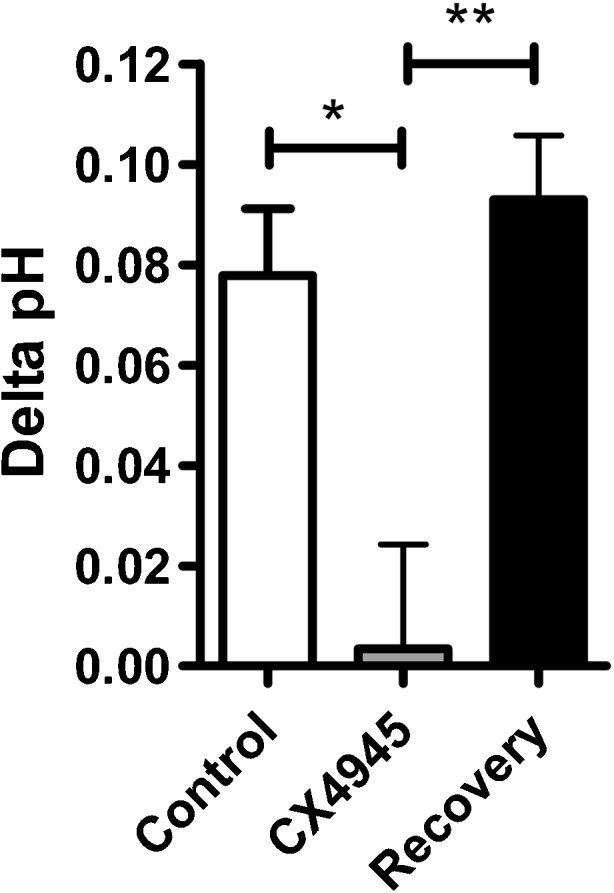
CK2 inhibition abolishes AE2 activity in primary human nasal epithelia: Well-differentiated, primary human nasal epithelia were isolated and cultured as described in the ‘Methods’ section. The activity of the basolateral Cl^−^/HCO_3_^−^ exchanger was assessed by measuring pH_i_ changes in response to replacement of basolateral Cl^−^ with gluconate. The effect of CX4945 treatment (10 μM; 5 min) and reversibility on the mean change in pH_i_ caused by basolateral Cl^−^ removal is shown. Note that in these experiments, because the change in pH_i_ induced by removal of basolateral Cl^−^ was relatively small, it was difficult to obtain accurate rates of reacidification after Cl^−^ readdition, particularly for CK2-treated cells, and therefore these data have not been included. *Significant effect of CX4945 treatment vs. control (*p* < 0.05); **Significant effect of CX4945 wash off (recovery) vs. CX4945 (*p* < 0.01). Data represents mean ± S.E.M., *n* = 4–5 from two donors

Having demonstrated that human AE2 was regulated by CK2 in a human airway epithelial cell line, we next assessed whether AE2 activity showed similar CK2-dependent regulation in well-differentiated human nasal epithelial (HNE) cultures. AE2 mRNA expression has previously been identified in the proximal airways and in HNE cells [1, 12, 55], and HNE cells have also been shown to possess a basolateral, DIDS-sensitive Cl^−^/HCO_3_^−^ exchanger, indicative of functional expression of AE2 [55]. To this end, intracellular pH measurements were performed on HNE monolayers and the effect of CK2 inhibition on AE2 activity was assessed. In control conditions, removal of basolateral Cl^−^ increased pH_i_ by 0.08 ± 0.01 pH_i_ units, and this response was significantly reduced to 0.01 ± 0.03 pH_i_ units in the presence of CX4945 (*n* = 5; *p* < 0.05; Fig. 8). The effect of CX4945 was reversible as the response to basolateral Cl^−^ removal could be recovered after wash out of the drug (0.09 ± 0.01 pH_i_ units; *n* = 4; *p* < 0.01 vs. CX4945; Fig. 8). Therefore, these data indicate that basolateral AE2 activity in primary human nasal epithelia is also positively regulated by CK2 which is consistent with our results from Calu-3 cells.

### Mouse AE2 displays identical regulation to the basolateral Cl^−^/HCO_3_^−^ exchanger in Calu-3 cells under resting conditions

**Fig. 9:**
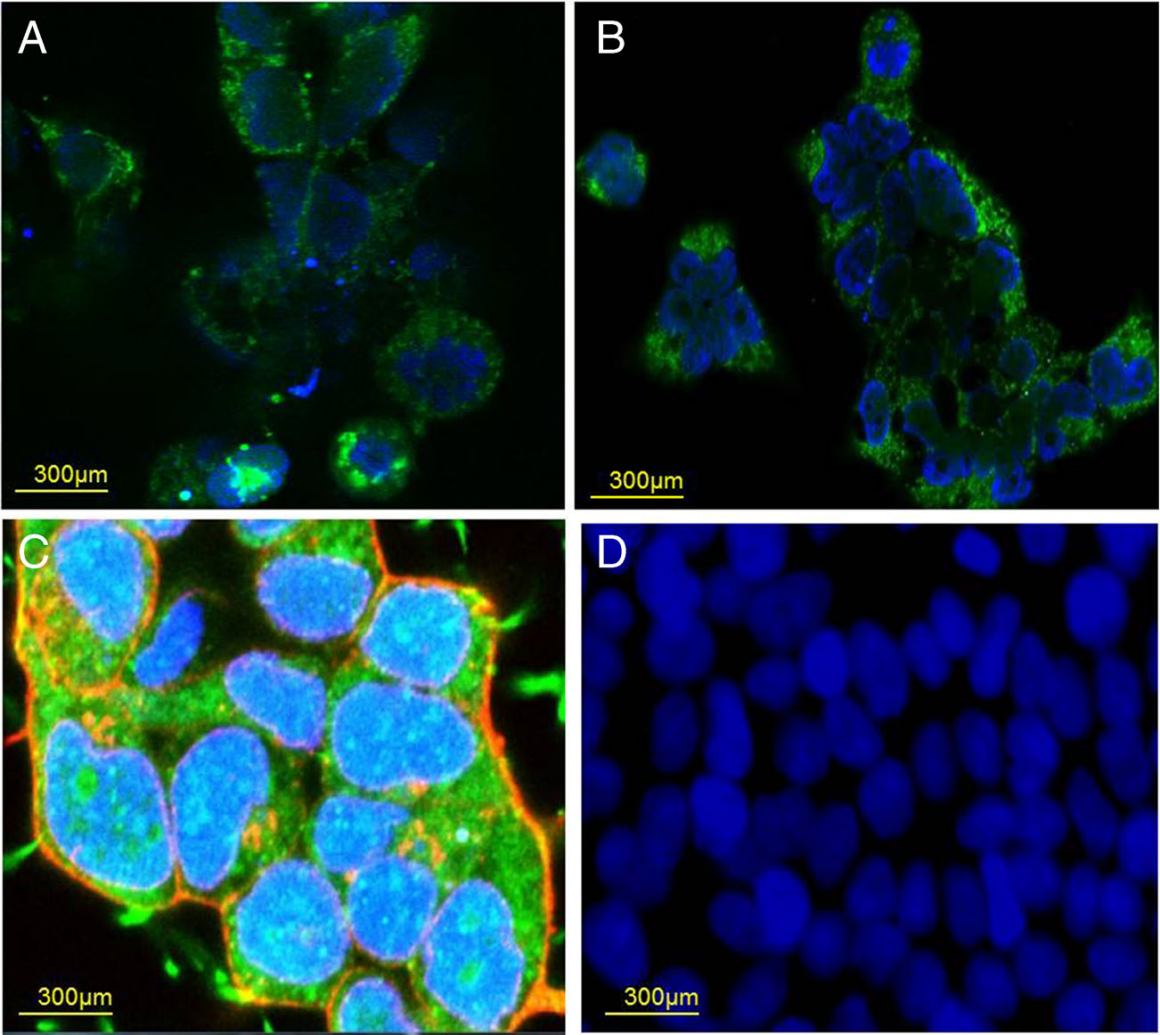
Expression and localization of mAE2 transiently transfected into HEK-293T cells: HEK-293T cells were transfected with HA-tagged mAE2 cDNA and studied 2 days post-transfection. **a** and **b** show confocal images in which mAE2 was labelled using a mouse anti-HA primary antibody and a goat anti-mouse secondary antibody conjugated to FITC fluorophore (green fluorescence). The nuclei were stained with DAPI (blue fluorescence). Significant intracellular as well as plasma membrane staining was observed. In **c,** the plasma membrane was stained with WGA (red fluorescence), and plasma membrane localisation of mAE2 was thus confirmed by yellow staining. **d** Shows non-transfected cells stained with mouse anti-HA primary antibody and goat anti-mouse FITC-conjugated secondary antibody. Data representative of three independent experiments

**Fig. 10:**
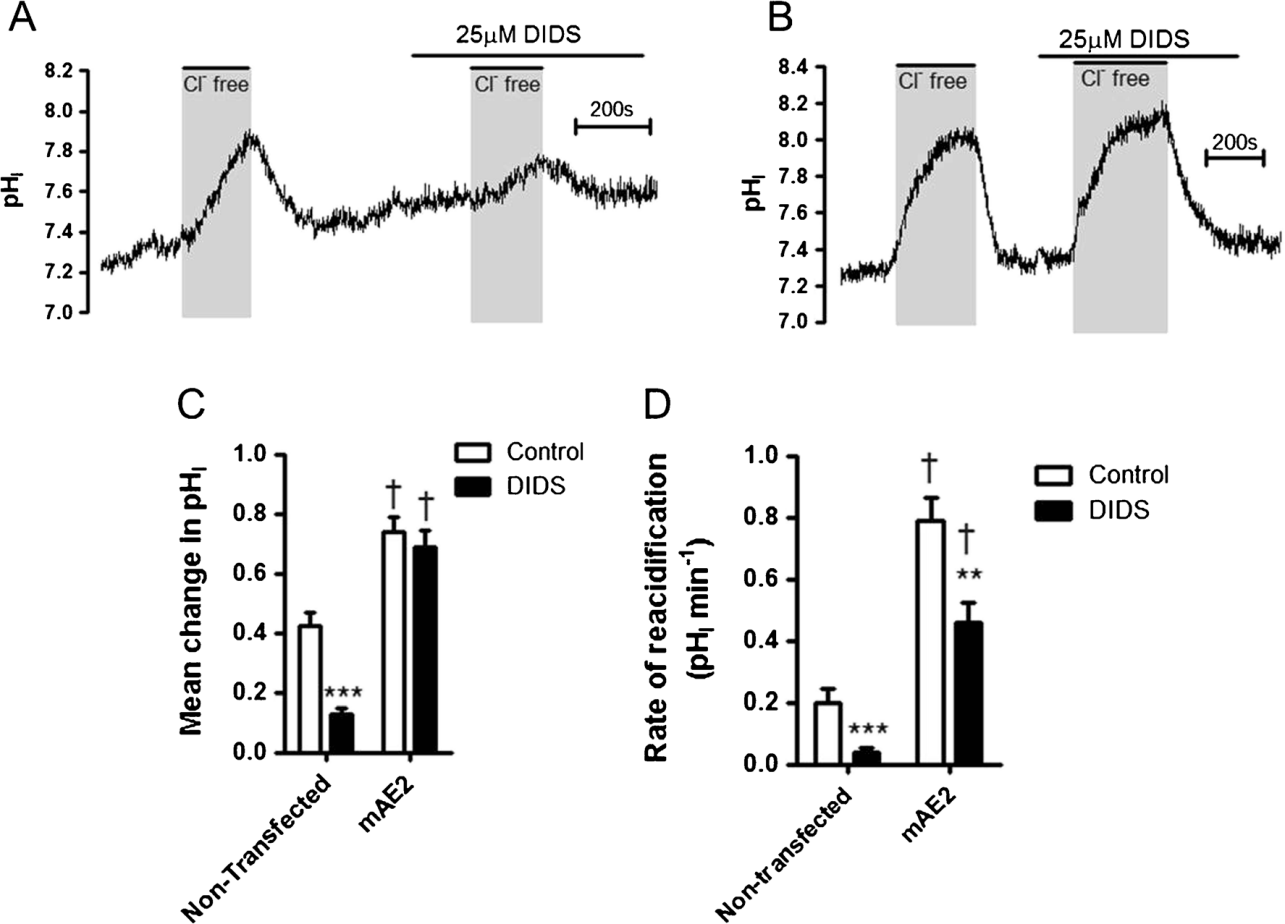
mAE2-expressing cells demonstrate enhanced Cl^−^/HCO_3_^−^ exchange activity and reduced sensitivity to DIDS compared to non-transfected cells: HEK-293T cells were transfected with HA-tagged mAE2 cDNA and studied 2 days post-transfection, and results compared to non-transfected cells. Cells were loaded with BCECF-AM (10 μM; 10 min) and pH_i_ was determined by fluorescent microscopy. The activity of Cl^−^/HCO_3_^−^ exchangers was assessed by measuring pH_i_ changes in response to replacement of extracellular Cl^−^ with gluconate. **a** and **b** show representative experiments in which Cl^−^/HCO_3_^−^ exchanger activity was measured in non-transfected cells and mAE2-transfected cells, respectively. **c** and **d** summarize the effect of both mAE2 transfection and DIDS (25 μM) on the mean change in pH_i_ caused by extracellular Cl^−^ removal and the rate of reacidification upon Cl^−^ readdition, respectively. **Significant effect of DIDS (*p* < 0.01; *** = *p* < 0.001); †Significant effect of mAE2 transfection (*p* < 0.01). Data represents mean ± S.E.M., *n* = 4 for non-transfected cells and *n* = 10 for transfected cells

The current data demonstrated the presence of a DIDS-sensitive Cl^−^/HCO_3_^−^ exchanger on the basolateral membrane of Calu-3 cells which was negatively regulated by cAMP, and positively regulated by Ca^2+^/CaM and CK2. Furthermore, CK2-regulation of a basolateral Cl^−^/HCO_3_^−^ exchanger was also demonstrated in well differentiated primary cultures of HNE cells. The properties of the exchanger suggested it was SLC4A2 (AE2). Therefore, to study the regulation of AE2 further, we expressed mouse AE2 (mAE2) in HEK-293T cells. Immunocytochemistry revealed successful transfection and expression of the protein at the cell surface of these cells (Fig. 9), although considerable intracellular expression was also observed. Intracellular pH measurements in non-transfected cells showed that HEK cells possessed an endogenous Cl^−^/HCO_3_^−^ exchange activity (as previously reported by Sterling et al. [58]). However, this endogenous AE activity was significantly reduced by a low concentration of DIDS (Fig. 10A), which had little effect on exogenously expressed mAE2 activity (Fig. 10B). Therefore, in all further experiments, mAE2 activity was assessed in the continuous presence of 25 μM DIDS. Using this approach, after 2 days of transfection, mAE2 expression increased both the magnitude of alkalinization in response to removal of basolateral Cl^−^ (0.13 ± 0.02 vs. 0.69 ± 0.06; *p* < 0.001; *n* = 10) and the rate of reacidification upon basolateral Cl^−^ readdition (0.04 ± 0.01 pH units min^−1^ vs. 0.46 ± 0.07 pH units min^−1^; *p* < 0.001; *n* = 10), compared to non-transfected cells (Fig. 10C, D, respectively). We found mAE2 activity to be sensitive to DIDS, with 100 μM DIDS causing a 40.0 ± 4.3% inhibition of the mean pH_i_ change in response to extracellular Cl^−^ removal (*p* < 0.001; *n* = 3) and a 80.4 ± 4.5% inhibition of the rate of reacidification in response to reintroduction of extracellular Cl^−^ (*p* < 0.001; *n* = 3).

**Fig. 11:**
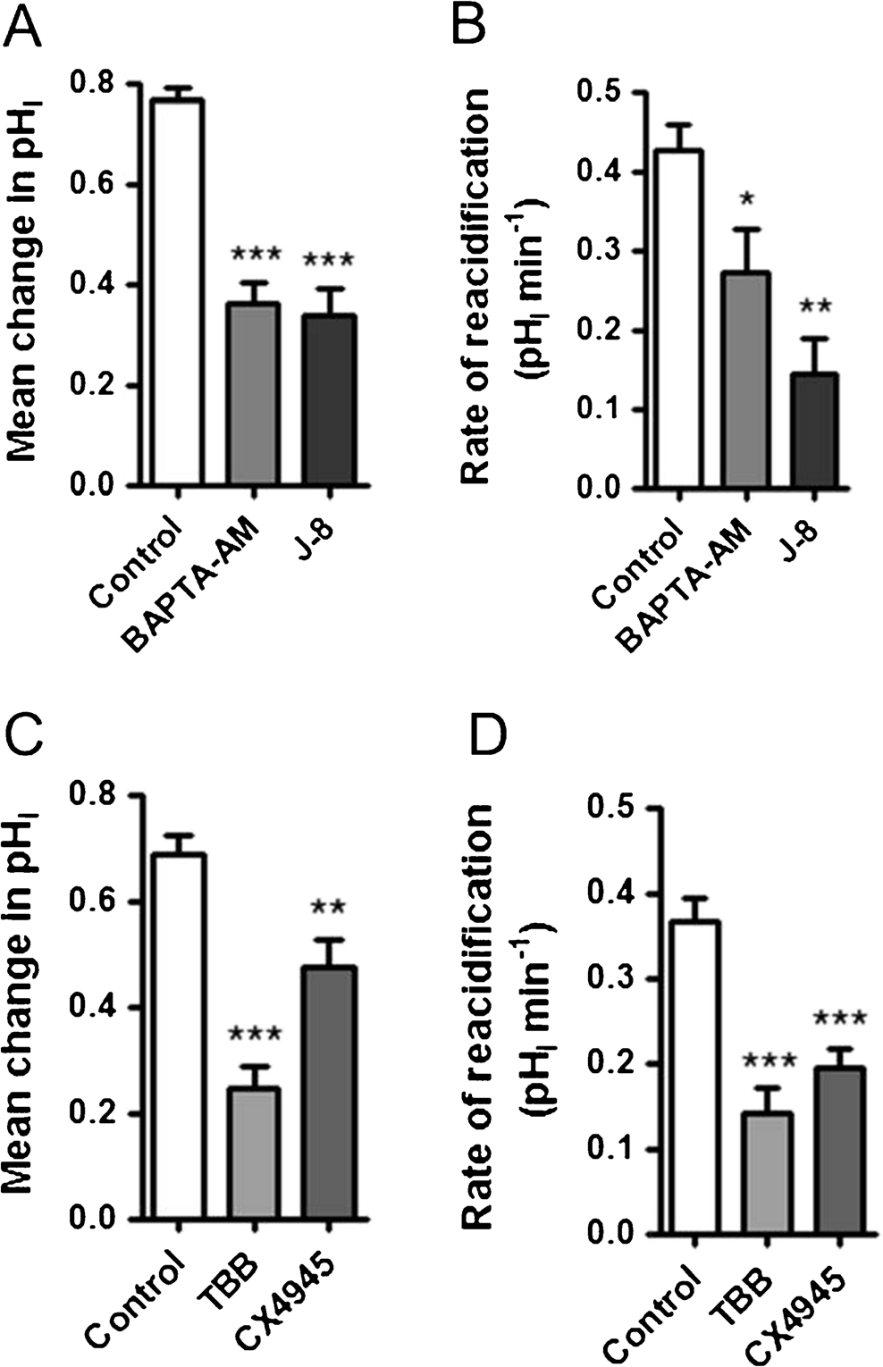
mAE2 is regulated by Ca^2+^/CaM signalling and by CK2: HEK-293T cells were transfected with HA-tagged mAE2 cDNA and studied 2 days post-transfection. Cells were loaded with BCECF-AM (10 μM; 10 min) and pH_i_ was determined by fluorescent microscopy. The activity of mAE2 was assessed by measuring pH_i_ changes in response to replacement of extracellular Cl^−^ with gluconate in the presence of 25 μM DIDS to inhibit endogenous Cl^−^/HCO_3_^−^ exchange activity. **a** and **b** display the effect of BAPTA-AM (50 μM) and J-8 (50 μM) on the mean change in pH_i_ caused by extracellular Cl^−^ removal and the rate of reacidification upon Cl^−^ readdition, respectively. *Significant effect of treatment vs. control (*p* < 0.05; ** = *p* < 0.01; *** = *p* < 0.001). Data represents mean ± S.E.M., *n* = 4–11. **c** and **d** display the effect of TBB (10 μM) and CX4945 (10 μM) treatment on the mean change in pH_i_ caused by extracellular Cl^−^ removal and the rate of reacidification upon Cl^−^ readdition, respectively. **Significant effect of treatment vs. control (*** = *p* < 0.001). Data represents mean ± S.E.M., *n* = 7–16

We next investigated whether mAE2 transiently expressed in HEK-293T cells was regulated in a similar fashion to human AE2 expressed in Calu-3 and HNE cells. Figure 11A–D shows that mAE2 activity in HEK-293T cells was reduced by both BAPTA-AM and J-8, as well as TBB and CX4945, demonstrating that both Ca^2+^/CaM signalling and CK2 regulate mAE2 when exogenously expressed in HEK-293T cells. In addition, short-term exposure to CX4945 also inhibited CK2 catalytic activity in HEK-293T cell lysates, demonstrating that the effect of CX4945 on mAE2 activity was very likely a result of CK2 inhibition (see Fig. 13B).

### Knockout of CK2 catalytic subunits reduces mAE2 activity in HEK-293T cells

**Fig. 12:**
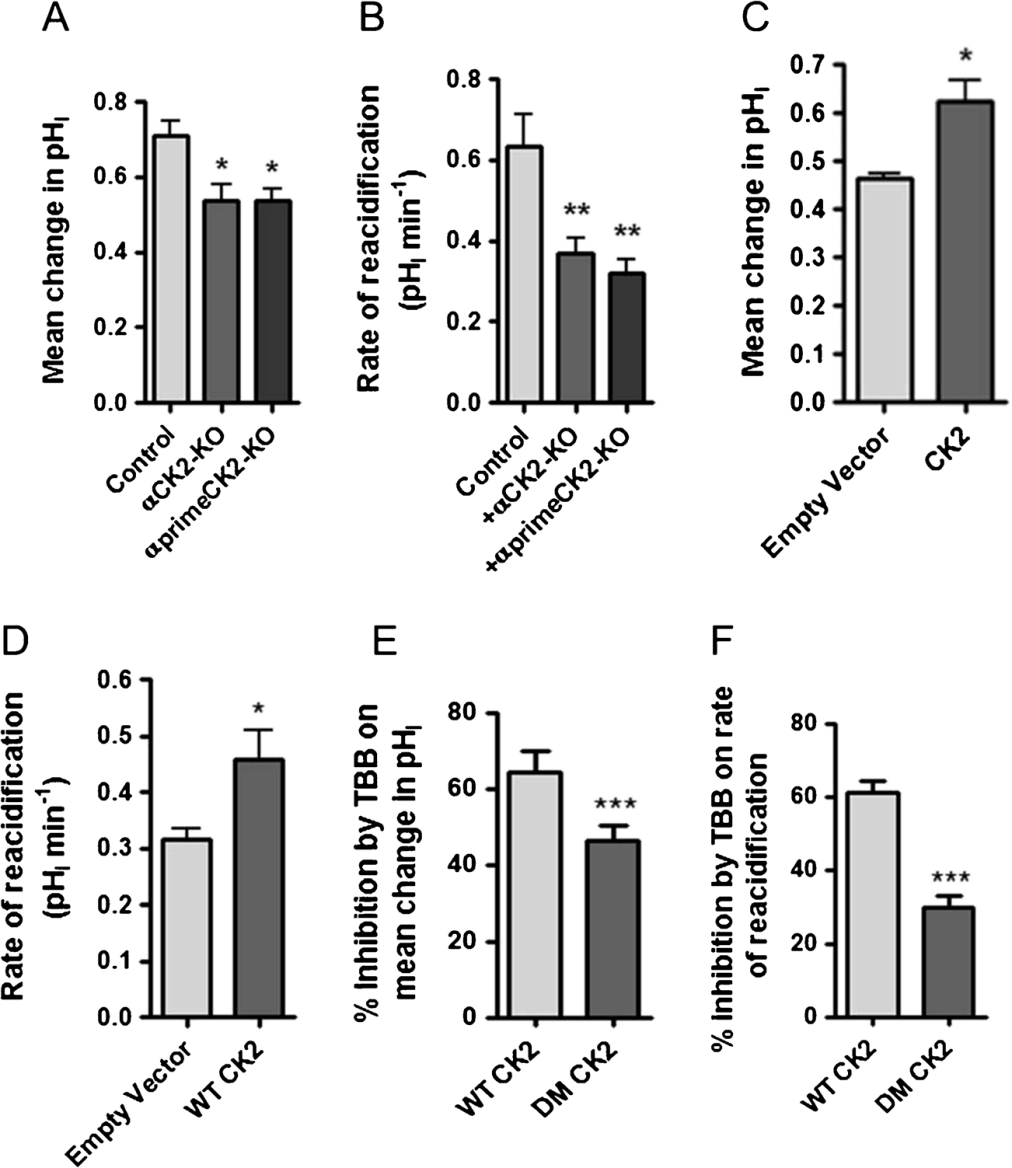
Genetic knockout of αCK2 reduces mAE2 activity which was rescued by reintroduction of αCK2 expression: **a** and **b** summarize the activity of mAE2 transiently expressed in either αCK2-KO HEK-293T cells or αprimeCK2-KO HEK-293T cells, assessed by measuring pH_i_ changes in response to replacement of extracellular Cl^−^ with gluconate in the presence of 25 μM DIDS to inhibit endogenous Cl^−^/HCO_3_^−^ exchanger activity. *Significant effect of αCK2 KO or αprimeCK2 KO (*p* < 0.05; ** = *p* < 0.01). Data represents mean ± S.E.M., *n* = 4–7. **c** and **d** summarize the activity of mAE2 transiently expressed in αCK2 KO HEK-293T cells that were also co-transfected with WT CK2. Anion exchanger activity was assessed by measuring pH_i_ changes in response to replacement of extracellular Cl^−^ with gluconate in the presence of 25 μM DIDS to inhibit endogenous Cl^−^/HCO_3_^−^ exchange activity. *Significant effect of WT CK2 expression (*p* < 0.05). Data represents mean ± S.E.M., *n* = 4. **e** and **f** display the percent inhibition of mAE2 activity by TBB in cells transfected with a CK2 double mutant (DM CK2). ***Significant difference between DM CK2 and WT CK2 (*p* < 0.001). Data represents mean ± S.E.M., *n* = 7–9

**Fig. 13:**
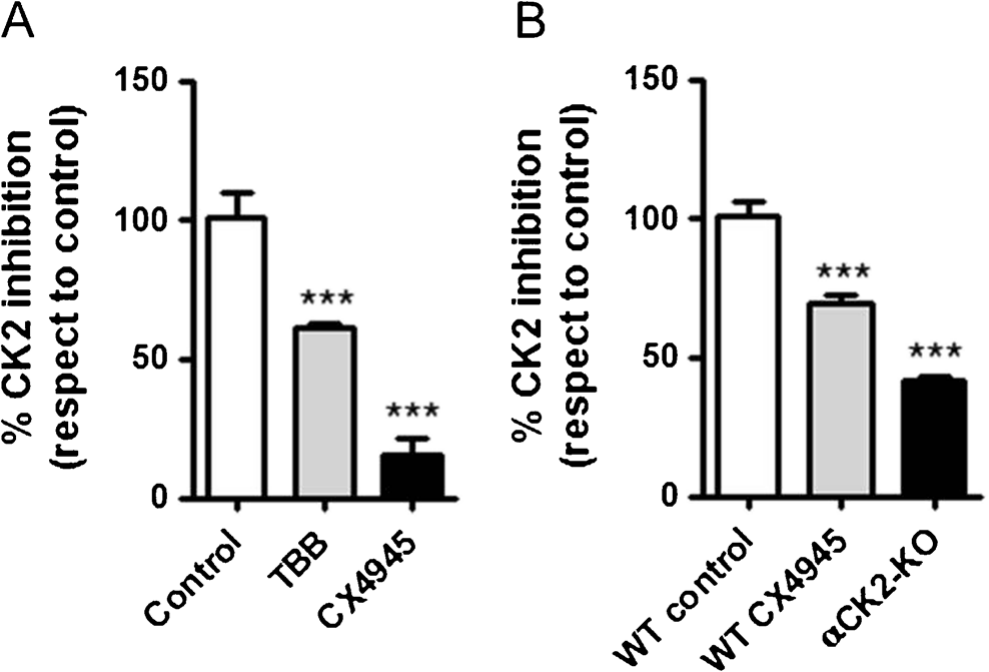
CK2 catalytic activity is inhibited by short-term exposure to specific inhibitors: Cell lysates were generated from **a** Calu-3 cells treated with TBB (10 μM; 5 min) or CX4945 (10 μM; 5 min) or **b** HEK-293T cells treated with CX4945 (10 μM; 5 min) or the αCK2-KO HEK-293T and CK2 activity was determined by means of radioactive assays with [γ-33P]ATP towards the specific CK2 substrate peptide CK2-tide (RRRADDSDDDDD).***Significant effect of inhibitor vs. untreated control or αCK2KO vs. control (*p* < 0.001). Data represents mean ± S.E.M., *n* = 4

To further investigate CK2 regulation of mAE2, we studied the activity of mAE2 in genetically altered HEK-293T cells. CK2 is a tetramer consisting of two catalytically active α subunits and two β subunits. We therefore genetically knocked out either the α catalytic subunit or the α prime catalytic subunit of CK2 (αCK2-KO and αprimeCK2-KO HEK-293T cells; see the ‘Methods’ section and supplementary Fig. 2). Note that α and α prime isoforms are highly homologous proteins with large overlapping functions and we found that CK2 catalytic activity was reduced by ~50% in αCK2-KO cells (Fig. 13B). In both types of CK2 KO cells, mAE2 activity was significantly reduced compared to WT HEK-293T cells, with the mean pH_i_ increase in response to removal of extracellular Cl^−^ reduced by 24.6 ± 6.4% in αCK2-KO cells (*p* < 0.05; *n* = 7; Fig. 12A) and 24.2 ± 4.0% in αprimeCK2-KO cells (*p* < 0.05; *n* = 6; Fig. 12A) while the rate of reacidification after extracellular Cl^−^ readdition was reduced by 42.0 ± 5.9% in αCK2-KO cells (*p* < 0.01; *n* = 7; Fig. 12B) and 49.4 ± 5.5% in αprimeCK2-KO cells (*p* < 0.01; *n* = 6; Fig. 12B). Furthermore, transfecting αCK2 KO HEK-293T cells with α-CK2 (to restore α CK2 activity) significantly recovered mAE2 activity in co-transfected cells, compared to ‘control’ co-transfected cells (i.e. mAE2 and empty plasmid) (Fig. 12C, D). Finally, co-transfecting normal HEK-293T cells with a double α catalytic CK2 mutant subunit with reduced sensitivity to TBB (DM CK2; see the ‘Methods’ section) [35, 65] led to a significant decrease in TBB inhibition of mAE2 activity, compared to control cells (Fig. 12E, F). Taken together with the pharmacological data, our results clearly indicate that CK2 plays a critical role in the regulation of mAE2 activity under resting conditions. These data are also consistent with the findings from Calu-3 cells and primary HNE cells, which provides further support that the identity of the basolateral Cl^−^/HCO_3_^−^ exchanger in airway epithelial cells is hAE2 (SLCA42). Thus, our results have uncovered a key regulatory mechanism for mammalian AE2 by CK2.

## Discussion

SLC4A2 has been identified on the basolateral membrane of human airway epithelia [1, 32] yet its regulation still remains poorly understood. Although the exact role of SLC4A2 in the conducting airways in general, including Calu-3 cells, still needs to be determined, it is likely to be important in regulating intracellular pH via its ability to transport HCO_3_^−^ across the basolateral membrane, as shown in this study and by others [14, 20, 24, 40]. Furthermore, because it transports Cl^−^ under resting conditions, AE2 will also act to accumulate Cl^−^ inside the cell, particularly when working in parallel with the sodium/potassium-dependent chloride (NKCC1) co-transporter that facilitates the influx of Na^+^, K^+^ and 2Cl^−^ ions across the basolateral membrane of Calu-3 cells [31]. Huang et al. [18] have shown AE2 knockdown in Calu-3 cells reduced transepithelial Cl^−^ secretion by ~60% whereas the NKCC1 inhibitor bumetamide reduced Cl^−^ secretion by ~20%. These results demonstrate the importance of AE2 in transepithelial Cl^−^ secretion. Here, we have explored the cellular mechanisms that regulate the activity of the basolateral Cl^−^/HCO_3_^−^ exchanger in Calu-3 cells.

We demonstrated that the IC_50_ for DIDS inhibition of the basolateral Cl^−^/HCO_3_^−^ exchanger was approximately 17 μM, which is in good agreement with the IC_50_ of ~13 μM reported by Humphreys et al. [19] for DIDS inhibition of human AE2 heterologously expressed in *Xenopus oocytes*. However, given that 500 μM DIDS was required to fully inhibit the exchanger suggested that other DIDS-sensitive transporters might also be present in the basolateral membrane of Calu-3 cells. This could be other SLC4 family members, such as SLC4A9 (AE4) which has recently been demonstrated in mouse submandibular gland acinar cells [41], or even members of the SLC26 family, such as SLC26A7, which play an important role in HCO_3_^−^ transport across the basolateral membrane of gastric parietal cells [42] and intercalated cells of the outer medullary collecting duct [43]. We observed that the basolateral Cl^−^/HCO_3_^−^ exchanger was active in non-stimulated Calu-3 cells yet elevations in cAMP, using a variety of different mechanisms, (i.e. activation of transmembrane adenylyl cyclase, inhibition of phosphodiesterases or inhibition of MRP-dependent cAMP efflux) all significantly inhibited the exchanger, demonstrating that cAMP negatively regulates this anion transporter. This cAMP-dependent inhibition of AE2-dependent HCO_3_^−^ efflux at the basolateral membrane would enhance the efficiency of cAMP-stimulated, CFTR-dependent HCO_3_^−^ secretion in Calu-3 cells. However, the downstream mechanism underlying AE inhibition by cAMP appeared not to involve PKA, as it was insensitive to two different PKA inhibitors, nor did it involve a number of other well characterized cAMP-dependent/PKA-independent signalling pathways, including EPAC and mTOR kinase. Although the present study supports our previous work [14], further investigations into the effect of cAMP on the basolateral Cl^−^/HCO_3_^−^ exchanger activity demonstrated that some forskolin-induced inhibition of the exchanger was alleviated in the presence of the CFTR inhibitor GlyH-101. These findings are similar, but not identical, to those reported by Huang et al. [18, 54] and Kim et al. [24] who failed to demonstrate any effect of cAMP on basolateral AE activity. These authors suggested that the apparent cAMP-dependent inhibition of the basolateral AE activity we observed was due to the activation of CFTR-dependent HCO_3_^−^ efflux across the apical membrane, which ‘swamped’ the basolateral response. Given that we were able to detect basolateral Cl^−^/HCO_3_^−^ exchanger activity in forskolin-stimulated cells when CFTR was inhibited lends support to this explanation. However, it is important to consider that the forskolin-stimulated inhibition of AE2 was not fully relieved, indicating there was still an effect of cAMP on the activity of the exchanger. In addition, our data, and those reported by others [18, 24, 54], do not explain how two different PKA inhibitors failed to affect the cAMP-dependent inhibition of the basolateral AE activity, particularly as we have previously shown these PKA inhibitors markedly reduced CFTR-dependent HCO_3_^−^ efflux in Calu-3 cells [14]. Clearly, further work is still required to unravel the role of cAMP in the regulation of AE2-dependent HCO_3_^−^ transport across the basolateral membrane of Calu-3 cells. It was also interesting that GlyH-101 alone affected the rate of reacidification caused by basolateral anion exchanger activity in the absence of cAMP stimulation (Fig. 3B). This effect is likely due to a rise in intracellular [Cl^−^], through an inhibition of Cl^−^ efflux by CFTR at the apical membrane, reducing the driving force for Cl^−^ entry at the basolateral membrane (on the reintroduction of external Cl^−^), and thereby reducing Cl^−^ /HCO_3_^−^ exchanger activity.

We also demonstrated that the exchanger was Ca^2+^-sensitive, as BAPTA-AM and depletion of thapsigargin-sensitive intracellular Ca^2+^ stores all markedly reduced the activity of the exchanger. One possible explanation for the effect seen with Ca^2+^ store depletion could involve a rise in cAMP, via the activation of store-operated cAMP signalling (socAMPs) as described by Lefkimmiatis et al. [30], in which the endoplasmic reticulum Ca^2+^-sensor STIM couples ER Ca^2+^ levels to cAMP production, via activation of transmembrane adenylyl cyclase 8. However, it is worth noting that in these intracellular Ca^2+^-depleted conditions, we observed that cAMP-stimulated Cl^−^/HCO_3_^−^ exchange at the apical membrane was also reduced which argues against socAMPs being activated by these conditions in Calu-3 cells (unpublished observations). In addition, inhibition of CaM using J-8 caused a marked reduction in the basolateral Cl^−^/HCO_3_^−^ exchanger activity under resting conditions, suggesting Ca^2+^-dependent activation of CaM was important for exchanger activity. To further understand the mechanism of CaM-dependent regulation of the basolateral Cl^−^/HCO_3_^−^ exchanger under resting conditions, the role of CK2 was assessed since it has been shown that CK2 is an important regulator of CaM activity via phosphorylation of three physiological CK2-phosphorylation acceptor sites in this Ca^2+^-binding protein [3, 46]. Treatment of Calu-3 cells with two different selective CK2 inhibitors, TBB and CX4945, caused a significant decrease in basolateral Cl^−^/HCO_3_^−^ exchanger activity under resting conditions. Furthermore, when cells were treated simultaneously with J-8 and TBB, there was very little further decrease in Cl^−^/HCO_3_^−^ exchanger activity, compared to the presence of each inhibitor alone. These results indicate that CK2 regulates the activity of the basolateral Cl^−^/HCO_3_^−^ exchanger under resting conditions, potentially through a CaM-dependent mechanism, although the role of CaM requires further investigation Therefore, our findings have uncovered an important role for CK2/CaM and calcium in the regulation of Cl^−^/HCO_3_^−^ exchange in human airway epithelia.

We also demonstrated that CX4945 significantly reduced basolateral Cl^−^/HCO_3_^−^ exchanger activity in primary HNE cells that have previously been shown to express AE2 mRNA and a functional, DIDS-sensitive Cl^−^/HCO_3_^−^ exchanger [55]. It was interesting to observe that the changes in pH_i_ in response to removal of basolateral Cl^−^ were approximately fourfold lower in HNE cells compared to Calu-3 cells, suggesting that there is more robust expression of AE2 in Calu-3 cells. Although Shin et al. [55] demonstrated pH_i_ changes of ~0.4 pH_i_ units in response to removal of basolateral Cl^−^, their experiments were performed on cells that had not fully differentiated. Indeed, in the same study, they demonstrated that AE2 expression decreased as cells differentiated and this likely underlies the different responses seen in their study and the current study. Nevertheless, these findings demonstrate CK2 regulation of basolateral Cl^−^/HCO_3_^−^ exchange also occurs in primary airway epithelial cultures which provide support for CK2 regulation of AE2 in vivo.

To strengthen our hypothesis that the DIDS-sensitive, Cl^−^/HCO_3_^−^ exchanger present on the basolateral membrane of human airway epithelia was SLC4A2 (AE2), we overexpressed mouse AE2 in HEK-293T cells and compared its regulation to the findings from Calu-3 cells. Similar to the data obtained in Calu-3 cells, mAE2 activity in HEK-293T cells was also reduced by chelation of intracellular Ca^2+^ and inhibition of CaM. This is consistent with findings from Chernova et al. [8] who showed that both BAPTA-AM and the CaM inhibitor, calmidazolium, significantly reduced Cl^−^ transport by murine AE2, when expressed in Xenopus oocytes, findings also reported by Stewart et al. [60]. Collectively, these results indicate that under resting conditions, CaM maintains normal AE2 activity potentially through a Ca^2+^-dependent pathway. Finally, we also demonstrated that pharmacological inhibition, or genetic knockout of CK2, significantly reduced mAE2 activity, consistent with results from Calu-3 and HNE cells. Because CK2 catalytic activity was reduced by ~50% in HEK-293T KO cells (Fig. 13B) and that mAE2 activity was reduced by a similar amount, (Fig. 12B) provides further evidence that CK2 plays a major role in the regulation of AE2 in airway epithelial cells. It was interesting to observe that the degree of inhibition by CX4945 was significantly less for mAE2 expressed in HEK-293T cells compared to that observed for the exchanger in Calu-3 cells (compare Figs. 6 with 11). This could be explained by the ‘addiction’ phenomenon (reviewed by Venerando et al. [66]) occurring in tumour cell lines, such as Calu-3, in which the level of CK2 is higher than in normal cells such as HEK-293T. Consequently, highly specific inhibition of CK2 by CX4945 in Calu-3 cells causes more pronounced downstream effects than those observed in HEK-293T cells. An interesting result in support of this hypothesis is shown in Fig. 13, where the percent inhibition of CK2 by CX4945 is significantly higher in Calu-3 cells compared to HEK-293T cells (69.6 ± 1.6% vs. 15.4 ± 3.0%; *p* < 0.001; *n* = 4; compare Fig. 13A, B), indicating Calu-3 cells have more active CK2 than HEK-293T cells.

In summary, we have demonstrated for the first time that human airway epithelial cells express a basolateral DIDS-sensitive, Cl^−^/HCO_3_^−^ exchanger which is regulated by CK2. Since mouse slc4A2 was regulated in an identical way when studied in a heterologous expression system suggests that the identity of the transporter in human airway cell was SLC4A2. Therefore, our findings identify CK2 as a new regulator of SLC4A2-dependent anion transport in human airway epithelia and suggest that CK2-dependent phosphorylation of SLC4A2, or an associated regulatory protein such as CaM, is essential for AE2 activity under resting conditions. We suggest that maintenance of basal AE2 activity will ensure efficient Cl^−^ loading into the cell prior to cAMP-stimulated anion secretion, which will help support net transepithelial HCO_3_^−^ and fluid secretion in airway epithelial cells [18]. Indeed, since CK2 also positively regulates the activity of CFTR suggests that this protein kinase plays an essential role in coordinating the activity of HCO_3_^−^ transporters at both the apical and basolateral membranes of airway epithelial cells.

## Electronic supplementary material

Supplementary Figure 1 Human and mouse AE2 contain CK2 phosphorylation sites. (A) shows the minimum canonical consensus sequence for CK2 phosphorylation. CK2 is an acidophilic Ser/Thr protein kinase that phosphorylate serine or threonine (S/T, in red) followed by glutamic or aspartic acid as well as pre-phosphorylated serine or threonine residues [47]. “x” represents any other residue but, as delineated by WebLogo3 analysis, there is a particular preference for acidic residues. (B) shows the alignment of human and mouse AE2 sequences. Already known phosphorylated sites are highlighted in red. CK2 consensus sequences on the basis of (A) are marked by squares. (DOCX 488 kb)

Supplementary Figure 2 CRISPR/Cas9 gene editing effectively knocks out the αCK2 and αprimeCK2 catalytic subunits in HEK-293T cells. CRISPR/Cas9 gene editing was performed on HEK-293T cells in order to knockout αCK2 and αprimeCK2 (for full details, see Methods). Cell lysates were made and 30 μg of protein was loaded on to 12% SDS-PAGE gels and expression of αCK2 and αprimeCK2 was analysed by Western Blotting using the indicated antibody. αCK2 antisera were raised in rabbit against the sequence of the human protein at the C-terminus [376–391], anti-αprimeCK2 was purchased from Santa Cruz Biotechnology (Santa Cruz, CA) and anti-β-actin was purchased from Sigma-Aldrich (Dorset, UK). The blot displays expression of αCK2, αprime CK2 and β-actin in WT, αCK2 knockout and αprimeCK2 knockout HEK-293T cells. Absence of a band corresponding to each protein confirmed successful knockout. (DOCX 488 kb)

## Abbreviations

AE2: Anion exchanger 2
ASL: Airway surface liquid
CK2: Casein kinase 2
PKA: Protein kinase A
