# Supplementary material for: CK2 is a key regulator of SLC4A2-mediated Cl−/HCO3− exchange in human airway epithelia
Source: Pflugers Arch. 2017 Apr 28;469(9):1073–91. doi: 10.1007/s00424-017-1981-3 (PMC5554290; doi:10.1007/s00424-017-1981-3)
Supplement: Supplementary file 1 — Human and mouse AE2 contain CK2 phosphorylation sites. (A) shows the minimum canonical consensus sequence for CK2 phosphorylation. CK2 is an acidophilic Ser/Thr protein kinase that phosphorylate serine or threonine (S/T, in red) followed by glutamic or aspartic acid as well as pre-phosphorylated serine or threonine residues [47]. “x” represents any other residue but, as delineated by WebLogo3 analysis, there is a particular preference for acidic residues. (B) shows the alignment of human and mouse AE2 sequences. Already known phosphorylated sites are highlighted in red. CK2 consensus sequences on the basis of (A) are marked by squares. (DOCX 488 kb) [file 424_2017_1981_MOESM1_ESM.docx]

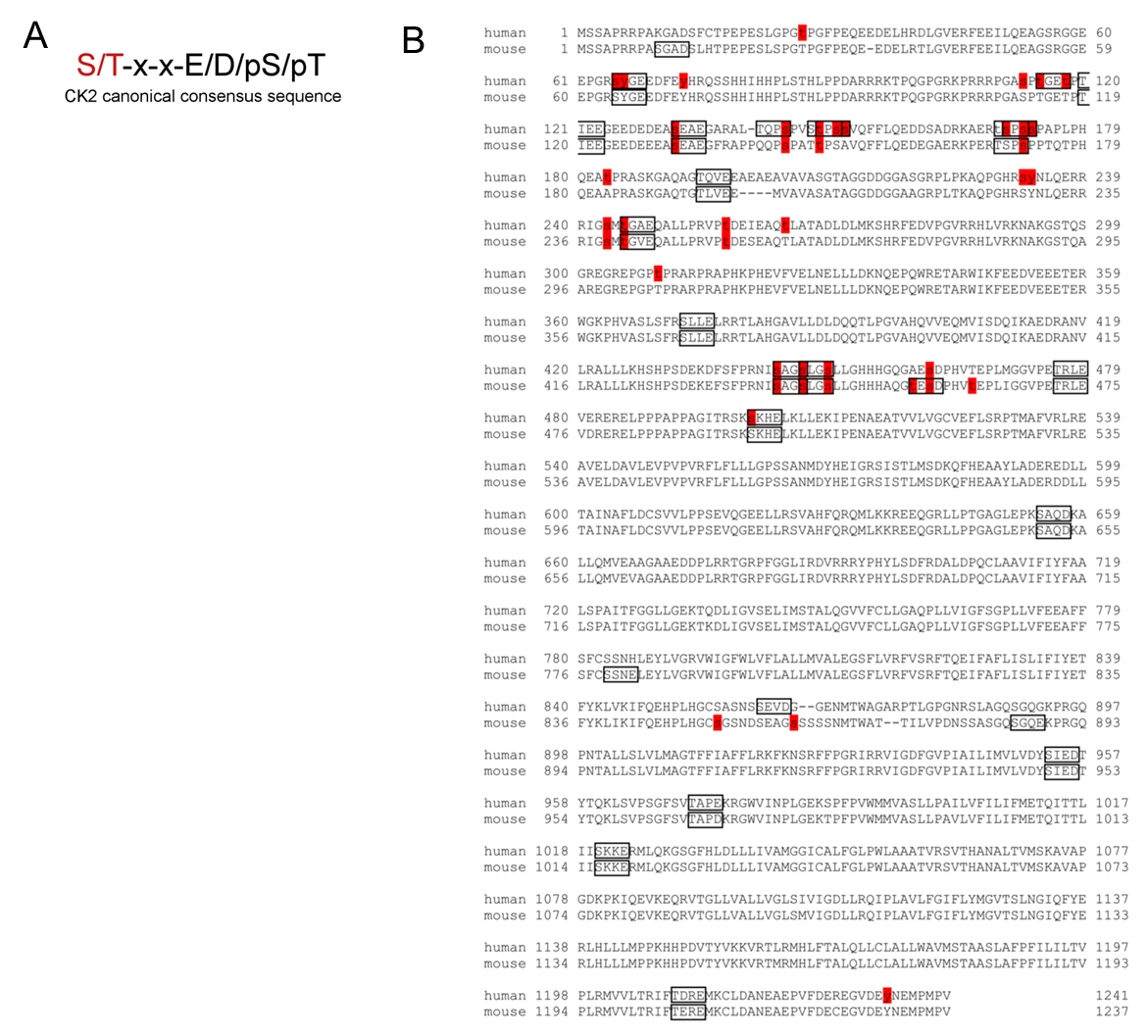


**Supplementary Figure 1: Human and mouse AE2 contain CK2 phosphorylation sites.** (A) shows the minimum canonical consensus sequence for CK2 phosphorylation. CK2 is an acidophilic Ser/Thr protein kinase that phosphorylate serine or threonine (S/T, in red) followed by glutamic or aspartic acid as well as pre-phosphorylated serine or threonine residues [64]. “x” represents any other residue but, as delineated by WebLogo3 analysis, there is a particular preference for acidic residues. (B) shows the alignment of human and mouse AE2 sequences. Already known phosphorylated sites are highlighted in red. CK2 consensus sequences on the basis of (A) are marked by squares.
