# Supplementary material for: CK2 is a key regulator of SLC4A2-mediated Cl−/HCO3− exchange in human airway epithelia
Source: Pflugers Arch. 2017 Apr 28;469(9):1073–91. doi: 10.1007/s00424-017-1981-3 (PMC5554290; doi:10.1007/s00424-017-1981-3)
Supplement: Supplementary file 2 — CRISPR/Cas9 gene editing effectively knocks out the αCK2 and αprimeCK2 catalytic subunits in HEK-293T cells. CRISPR/Cas9 gene editing was performed on HEK-293T cells in order to knockout αCK2 and αprimeCK2 (for full details, see Methods). Cell lysates were made and 30 μg of protein was loaded on to 12% SDS-PAGE gels and expression of αCK2 and αprimeCK2 was analysed by Western Blotting using the indicated antibody. αCK2 antisera were raised in rabbit against the sequence of the human protein at the C-terminus [376–391], anti-αprimeCK2 was purchased from Santa Cruz Biotechnology (Santa Cruz, CA) and anti-β-actin was purchased from Sigma-Aldrich (Dorset, UK). The blot displays expression of αCK2, αprime CK2 and β-actin in WT, αCK2 knockout and αprimeCK2 knockout HEK-293T cells. Absence of a band corresponding to each protein confirmed successful knockout. (DOCX 488 kb) [file 424_2017_1981_MOESM2_ESM.docx]

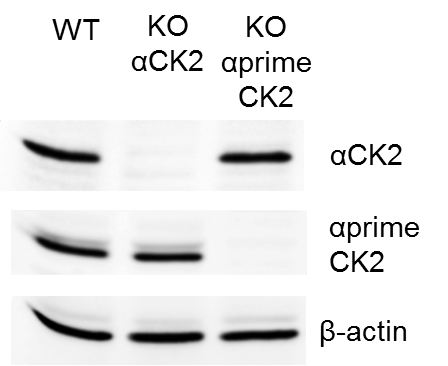


**Supplementary Figure 2: CRISPR/Cas9 gene editing effectively knocks out the αCK2 and αprimeCK2 catalytic subunits in HEK-293T cells.** CRISPR/Cas9 gene editing was performed on HEK-293T cells in order to knockout αCK2 and αprimeCK2 (for full details, see Methods). Cell lysates were made and 30 µg of protein was loaded on to 12% SDS-PAGE gels and expression of αCK2 and αprimeCK2 was analyzed by Western Blotting using the indicated antibody. αCK2 antisera were raised in rabbit against the sequence of the human protein at C-terminus [376–391], anti-αprimeCK2 was purchased from Santa Cruz Biotechnology (Santa Cruz, CA) and anti-β-actin was purchased from Sigma-Aldrich (Dorset, UK). The blot displays expression of αCK2, αprime CK2 and β-actin in WT, αCK2 knockout and αprimeCK2 knockout HEK-293T cells. Absence of a band corresponding to each protein confirmed successful knockout.
